# A Dual-Functional Orphan Response Regulator Negatively Controls the Differential Transcription of Duplicate *groEL*s and Plays a Global Regulatory Role in *Myxococcus*

**DOI:** 10.1128/msystems.01056-21

**Published:** 2022-03-30

**Authors:** Li Zhuo, Tian-yu Wan, Zhuo Pan, Jia-ning Wang, Duo-hong Sheng, Yue-zhong Li

**Affiliations:** a State Key Laboratory of Microbial Technology, Institute of Microbial Technology, Shandong University, Qingdao, People’s Republic of China; FMRP-USP

**Keywords:** two-component system, orphan response regulator, duplicate *groEL*s, differential transcription, *Myxococcus*

## Abstract

Differential transcription of functionally divergent duplicate genes is critical for bacterial cells to properly and competitively function in the environment, but the transcriptional regulation mechanisms remain in mystery. Myxococcus xanthus DK1622 possesses two duplicate *groEL*s with divergent functions. Here, we report that *MXAN_4468*, an orphan gene located upstream of *groEL2*, encodes a response regulator (RR) and is responsible for the differential expression regulation of duplicate *groEL*s. This RR protein realizes its negative regulatory role via a novel dual-mode functioning manner: binding to the transcription repressor HrcA to enhance its transcriptional inhibition of duplicate *groEL*s and binding to the 3′ end of the *MXAN_4468* sequence to specifically decrease the transcription of the following *groEL2*. Phosphorylation at the conserved 61^st^ aspartic acid is required to trigger the regulatory functions of MXAN_4468. Pull-down experiment and mutation demonstrated that two noncognate CheA proteins, respectively belonging to the Che8 and Che7 chemosensory pathways, are involved in the protein phosphorylation. A transcriptome analysis, as well as the pull-down experiment, suggested that MXAN_4468 plays a global negative regulatory role in M. xanthus. This study elucidates, for the first time, the regulatory mechanism of differential transcription of bacterial duplicate *groEL*s and suggests a global regulatory role of a dual-functional orphan RR.

**IMPORTANCE** Multiply copied *groEL*s require precise regulation of transcriptions for their divergent cellular functions. Here, we reported that an orphan response regulator (RR) tunes the transcriptional discrepancy of the duplicate *groEL*s in Myxococcus xanthus DK1622 in a dual-functional mode. This RR protein has a conserved phosphorylation site, and the phosphorylation is required for the regulatory functions. Transcriptomic analysis, as well as a pull-down experiment, suggests that the RR plays a global regulatory role in M. xanthus. This study highlights that the dual-functional orphan RR might be involved in conducting the transcriptional symphony to stabilize the complex biological functions in cells.

## INTRODUCTION

Two-component systems (TCSs) serve as the basic stimulus-response coupling mechanism allowing bacterial cells to sense and respond to diverse changes in environment (1, 2). Typically, a TCS consists of a histidine kinase (HK) and a cognate response regulator (RR). HK undergoes an ATP-dependent autophosphorylation of a conserved histidine (His) residue upon stimulation, creating a high-energy phosphoryl group that is subsequently transferred to the aspartate (Asp) residue of the downstream RR. Then the phosphorylated RR activates or represses transcription of target genes (1, 3). Although paired HK and RR may function efficiently, orphan RR is universal in bacteria in a broad range of numbers and ratios For instance, 5 of the 32 RRs in Escherichia coli are orphan (4), 2 of the 20 RRs in Streptococcus sobrinus are orphan (5), and 2 of the 16 RRs in Acinetobacter baumannii are orphan (6). In comparison, Myxococcus xanthus possesses a total of 119 RRs, and more than 50% of them are orphan (7). During the past decade, functions of orphan RRs have been investigated in some bacteria, for example, in *Streptomyces* (8). However, little is known about the regulatory mechanisms of orphan RRs.

GroEL is an important molecular chaperone that belongs to the Hsp60 family of heat shock proteins, and it is involved in diverse biological functions in bacterial cells by participating in folding, maturation, and transport of many proteins under normal growth or heat shock conditions (9, 10). Most bacteria have a single copy of *groEL*, but the presence of two or more copies has been identified in 19.5% of the sequenced bacterial genomes (11). Duplicate *groEL*s are strictly transcribed at different levels, to fit their divergent cellular functions. For example, M. xanthus DK1622 has duplicate *groEL*s: *groEL1* plays an essential role in development and sporulation, while *groEL2* is required for cell predation and biosynthesis of the secondary metabolite myxovirescin (12–14). The transcriptional levels of these two *groEL*s are significantly different: the transcriptional level of *groEL1* is normally four times that of *groEL2*, and the transcriptional level of single *groES*, which is required for the functions of duplicate *groEL*s, is almost the sum of the *groEL1* and *groEL2* transcriptional levels (11). We previously reported that the global positive regulator σ^32^ and the local negative regulator HrcA are involved in the transcriptional regulation; these two regulators respectively target the −10/−35 region and the CIRCE element, which are separate in the promoter of *groEL1* but overlapped in that of *groEL2* (15). However, the regulation of σ^32^ and HrcA does not explain the strictly differential transcription of duplicate *groEL*s.

In M. xanthus DK1622, the *groEL1* and *groEL2* genes have distinct compositions and locations on the genome; *groEL1* (*MXAN_4895*) is located in a *groESL* operon, while *groEL2* (*MXAN_4467*) has no neighboring *groES* and is downstream of *MXAN_4468*, which encodes an orphan RR. In this study, we found that *MXAN_4468* deletion eliminated the negative transcriptional control of both *groEL*s and increased the expression of *groEL2* to almost the same level as that of *groEL1*. The RR has a conserved phosphorylation site, and phosphorylation of this Asp residue is required to trigger the RR regulatory functions. Pull-down assay and mutation indicated that two CheA proteins might be the noncognate HKs of this orphan RR. We determined that the regulator plays a central role for differential expression of duplicate *groEL*s in a dual-functioning mode: it enhances the ability of HrcA to bind to the CIRCE sequences to regulate the transcription of *groEL1* and *groEL2*, and it binds to the 3′ end of its own gene sequence to specifically negatively control the transcription of the downstream *groEL2* gene. A network analysis based on the transcriptome data, as well as the pull-down experiment, suggested that the RR gene plays a global negative regulatory role and primarily affects the transcription of stress regulatory proteins. This study highlights that this orphan RR in M. xanthus cells negatively regulates the transcription of duplicate *groEL*s and is involved in the control of complex biological functions.

## RESULTS

### The RR gene upstream of *groEL2* is a negative regulator of the transcription of duplicate *groEL*s in Myxococcus xanthus.

**FIG 1:**
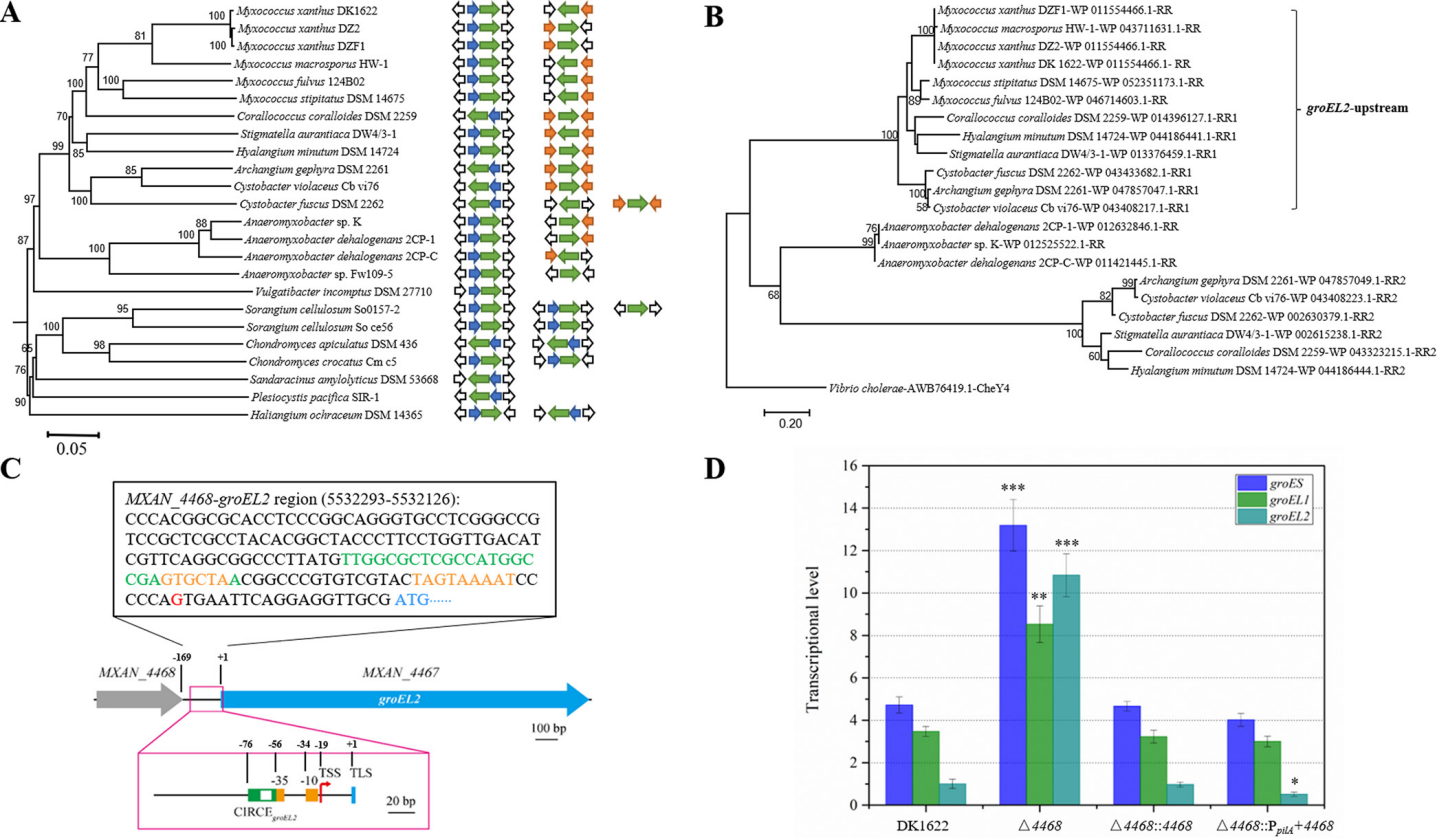
The RR genes in myxobacteria. (A) The composition and organization of the *groEL*, *groES*, and RR genes in the 24 sequenced myxobacterial genomes. The green, blue, and orange arrows show the transcription direction of the *groEL*, *groES*, and RR genes, respectively. (B) Evolutionary relationship analysis of myxobacterial RR neighboring *groEL*s. Vibrio cholerae chemotaxis-associated protein CheY4 was used as the outroot. (C) Representation of the region of *groEL2* and *MXAN_4468* in M. xanthus DK1622. The bar in the panel equals a 20-bp distance. (D) qPCR analysis of the transcriptional levels of *groES* and *groEL*s in *MXAN_4468* knockout, complementation, and overexpression mutants and the wild-type strain DK1622 after 24 h of incubation. The transcriptional level of *groEL2* in DK1622 was set to 1. For statistical analysis, “***,” “**,” and “*” indicate *P* values of <0.001, <0.01, and <0.05, respectively.

M. xanthus DK1622 has two *groEL*s and one *groES*; *groEL1* forms a complete operon with the single *groES*, and *groEL2* exists alone. *MXAN_4468* lies upstream of *groEL2* and is annotated by NCBI to encode a response regulator (RR) belonging to the cdd388505 protein family. *MXAN_4468* is an orphan RR. We found that the RR gene is located adjacent to *groEL2*, upstream and/or downstream, in a phylogenetically specific manner in different myxobacteria but does not occur adjacent to *groEL1* (Fig. 1A; see also Table S1 in the supplemental material). Based on the sequenced myxobacterial genomes, the *MXAN_4468* homologues locate upstream of *groEL2* in the same direction in *Myxococcus* genomes, downstream of *groEL2* in the opposite direction in *Anaeromyxobacter* genomes, and upstream and downstream of *groEL2* in the genomes of the genera *Corallococcus*, *Stigmatella*, and *Cystobacter*. However, there is no RR gene adjacent to *groEL2* in the genomes of the *Sorangineae* suborder (Fig. 1A). The RR proteins encoded by the upstream genes are highly conserved, the second RR proteins encoded by the downstream RR genes are closely grouped in a separate branch, and the single downstream RRs in the *Anaeromyxobacter* genomes form the third branch (Fig. 1B). These phylogenetically conserved RR genes might have similar cellular functions critical for myxobacteria.

10.1128/mSystems.01056-21.7 TABLE S1 Occurrences of cdd388505 protein family encoding genes adjacent to *groEL*s in sequenced myxobacteria and cdd388505-encoding genes in M. xanthus DK1622. Download Table S1, DOCX file, 0.03 MB. Copyright © 2022 Zhuo et al. 2022 Zhuo et al. https://creativecommons.org/licenses/by/4.0/ This content is distributed under the terms of the Creative Commons Attribution 4.0 International license.

The *MXAN_4468* and *groEL2* genes are separated by a 169-bp sequence, and the two genes were confirmed to be transcribed by their own promoters (Fig. 1C and Fig. S1A). This interval sequence contains the core promoter region of *groEL2*, which includes a CIRCE sequence for binding the negative regulator HrcA (15). We found that deletion of the RR gene greatly increased the transcriptional levels of *groES*, *groEL1*, and *groEL2* in DK1622 (Fig. 1D; *t* test, *groES* and *groEL2*, *P* value < 0.001; *groEL1*, *P* value < 0.01). The increases in the transcriptional levels of *groES* and *groEL*s were completely abolished by the *in situ* complementation of *MXAN_4468*. However, *in situ* overexpression of *MXAN_4468* had no effect on the transcription of *groES* and *groEL1* but significantly decreased *groEL2* expression (Fig. 1D; *t* test, *P* value < 0.05). The RR gene deletion, complementation, and overexpression were confirmed by quantitative PCR (qPCR) analysis (Fig. S1B). The above-described results indicate that *MXAN_4468* is a strong negative regulator of the expression of *groES* and *groEL*s and has an additional inhibitory effect specifically on *groEL2*. The transcriptional curves of *groES* and *groEL*s in the *MXAN_4468* knockout strain indicated that compared with wild-type DK1622, the negative regulation of *groES* and *groEL*s by the *MXAN_4468* gene occurred mainly in the exponential growth phase, when the chaperonin genes were expressed under either the normal temperature condition (30°C) or after heat shock at 42°C for 1 h (Fig. S1C). Notably, according to the transcriptome data, the transcription of *MXAN_4468* was significantly changed at 24 h and 36 h under the normal and heat shock conditions (Fig. S1D).

10.1128/mSystems.01056-21.2 FIG S1 Transcriptional analysis of *MXAN_4468*. (A) RT-PCR detection of the cotranscription of *MXAN_4468* and *groEL2* in M. xanthus DK1622. Lines 1 and 4, PCR amplification of the *MXAN_4468-groEL2* locus using DK1622 cDNA as the template. The *MXAN_4468*-*groEL2* locus was amplified by two pairs of primers (P1 and P2). Lines 2 and 5, positive controls using the total DNA extracted from DK1622 as the template. Lines 3 and 6, negative controls in which no reverse transcriptase was added. Line 7, PCR amplification of *MXAN_4468* using DK1622 cDNA as the template. Line 8, PCR amplification of *groEL2* using DK1622 cDNA as the template. M, Trans 2K Plus II markers. (B) Quantitative PCR analysis of the *MXAN_4468* transcriptional level in the *MXAN_4468* knockout, the complement and overexpression mutants, and the wild-type strain of M. xanthus DK1622 after 24 h of incubation. The transcriptional level of *MXAN_4468* was set to 1. (C) Quantitative PCR analysis of the transcriptional levels of *groES* and *groEL*s in the *MXAN_4468* knockout mutant and the wild-type strain of M. xanthus DK1622 grown in CTT medium under the normal temperature condition (30°C) and after heat shock at 42°C for an additional 1 h at different time points. The transcriptional level of *groEL2* in DK1622 at 24 h (normal incubation time) was set to 1. (D) Transcriptome data of *MXAN_4468* in M. xanthus DK1622 under the normal or heat shock condition after 24 h or 36 h of incubation. FPKM, expected number of fragments per kilobase of transcript sequence per million base pairs sequenced. For statistical analysis, “***,” “**,” and “*” indicate *P* values of <0.001, <0.01, and <0.05, respectively. Download FIG S1, TIF file, 1.7 MB. Copyright © 2022 Zhuo et al. 2022 Zhuo et al. https://creativecommons.org/licenses/by/4.0/ This content is distributed under the terms of the Creative Commons Attribution 4.0 International license.

### The orphan MXAN_4468 is phosphorylated by two noncognate CheAs belonging to the Che8 and Che7 chemosensory pathways.

**FIG 2:**
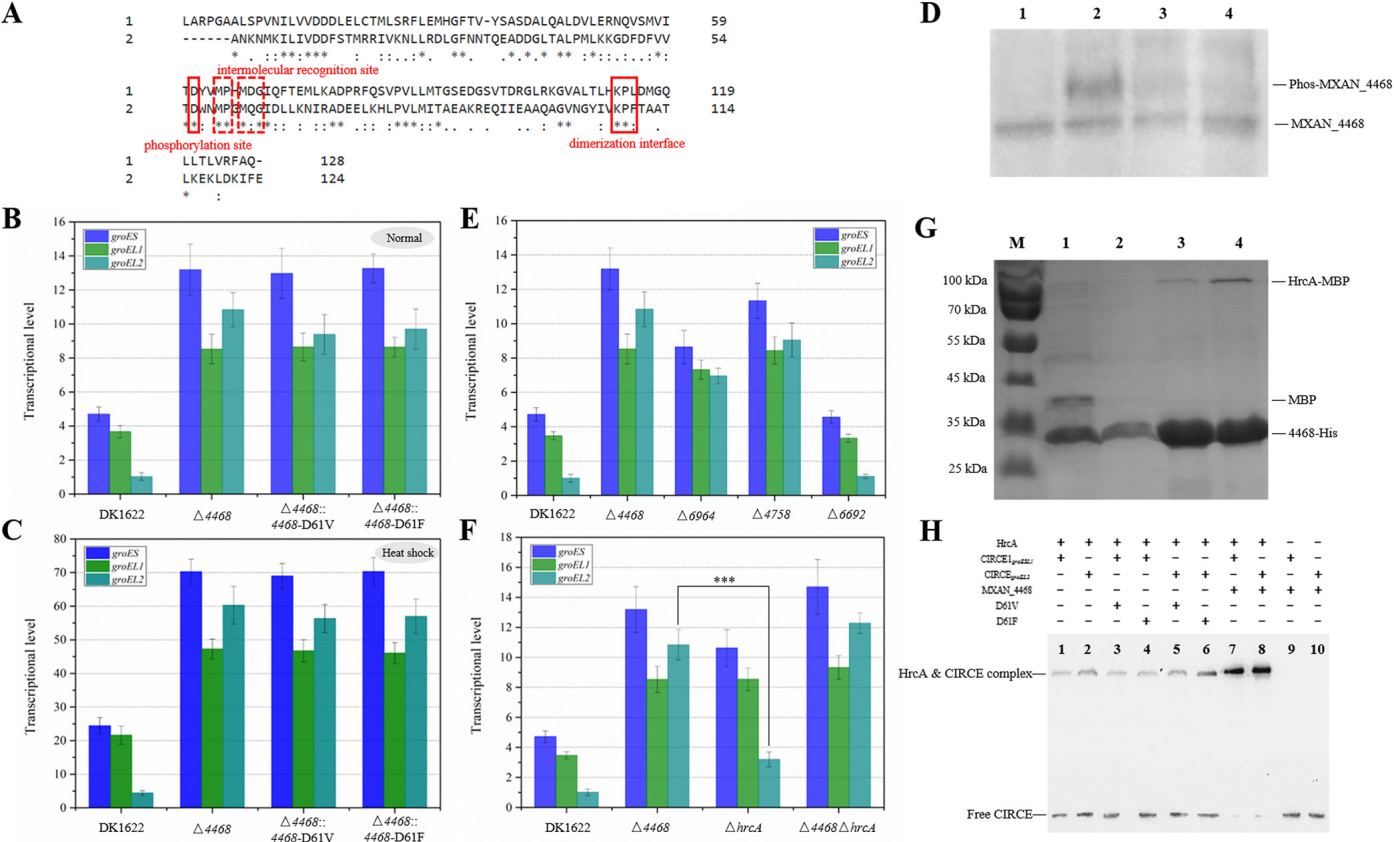
Phosphorylated MXAN_4468 binds to HrcA to inhibit the *groESL* transcription. (A) Bi-sequence alignment analysis of M. xanthus MXAN_4468 protein and Vibrio cholerae chemotaxis-associated protein CheY4. Alignments 1 and 2 are the amino acid sequences of M. xanthus MXAN_4468 and Vibrio cholerae CheY4, respectively. The red solid lines indicate phosphorylation sites and the dimerization interface, and the red dashed lines represent intermolecular recognition sites. (B and C) The transcriptional levels of *groES*, *groEL1*, and *groEL2* in M. xanthus DK1622 and *MXAN_4468* mutants grown in CTT medium at a normal temperature (B) and at heat shock for 1 h more (C). The transcriptional level of *groEL2* in the wild-type DK1622 under the normal condition was set to 1. (D) Phos-tag PAGE analysis of MXAN_4468 mutants. Lane 1, purified 4468-His protein cloned in E. coli; lane 2, Δ*4468*::*4468*-His wild-type strain; lane 3, Δ*4468*::*4468*-D61V-His mutant; lane 4, Δ*4468*::*4468*-D61F-His mutant. For all of the assay, no additional HK proteins were added. (E) qPCR analysis of the transcriptional levels of *groES* and *groEL*s in the *MXAN_4468* knockout mutant, the two *cheA* deletion mutants, the *MXAN_6692* knockout mutant, and wild-type strain DK1622 after 24 h of incubation. (F) qPCR analysis of the transcriptional levels of *groES* and *groEL*s in *MXAN_4468* and *hrcA* knockout mutants and the *MXAN_4468* and *hrcA* double-deletion mutant. For statistical analysis, “***” means *P* value of <0.001. The transcriptional level of *groEL2* in the wild-type DK1622 was set to 1 (E and F). (G) Pull-down analysis of MXAN_4468 and HrcA detected by SDS-PAGE. M, marker: PageRuler™ prestained protein ladder. Lane 1, MBP protein plus MXAN_4468-His protein penetrating liquid; lane 2, MBP protein plus MXAN_4468-His 250 mM imidazole elution product; lane 3, HrcA-MBP plus MXAN_4468-His penetrating liquid; lane 4, HrcA-MBP plus MXAN_4468-His 250 mM imidazole-elution product. (H) Effects of the addition of MXAN_4468 and MXAN_4468 mutant proteins (D61V and D61F) on the interaction between HrcA and CIRCE (presented by native PAGE). The components added in each lane are labeled at the top.

MXAN_4468 is a single-domain RR, which is represented by the chemotaxis-associated protein CheY (16). This protein has the highest sequence identity with CheY4 of Vibrio cholerae (42.48%), and the two proteins are also highly similar in structure based on the modeling analysis (Fig. S2A). CheY4 is involved in chemotaxis-related motility to control the direction of flagellar movement (17, 18). Active CheY4 is a phosphorylated protein, and the phosphorylated amino acid is located in the domain that receives signals transmitted from the upstream component (19). Based on the amino acid sequence alignment with CheY4, MXAN_4468 may contain a similar phosphorylation site at the 61^st^ aspartic acid (Fig. 2A). Notably, M. xanthus DK1622 has 20 single-domain RR homologues belonging to the cdd388505 protein family (Table S1), and some of them have been annotated in different chemosensory pathways (20, 21). These 20 RR homologues in M. xanthus DK1622 (Fig. S2B), as well as the 21 *groEL2*-adjacent RRs in different myxobacterial genomes (Fig. S2C), were all conserved at the 61^st^ aspartic acid.

10.1128/mSystems.01056-21.3 FIG S2 Homology modeling, WebLogo analysis, and purification of RR proteins. (A) Homology modeling of MXAN_4468 protein in M. xanthus DK1622. The N and C termini are shown in blue and red, respectively. The Vibrio cholerae chemotaxis-associated protein CheY4 is depicted by a purple line. TM = 0.909, which is greatly higher than the threshold of 0.5, suggesting the same folding with acceptable modeling accuracy of the two compared proteins. (B) WebLogo view of the alignment of the RR proteins in M. xanthus DK1622. The red box indicates the 61^st^ amino acid as the phosphorylation site. (C) Analysis of the conserved sites of RR proteins flanking *groEL2* in the sequenced genome of myxobacteria. The red solid lines indicate the phosphorylation site and the dimerization interface. The red dashed lines represent the intermolecular recognition sites. (D) Preparation and purification of MXAN_4468 and its mutant proteins with a His tag at the C terminus, showing that most purified proteins were in polymer forms. M, marker: PageRuler™ prestained protein ladder. Line 1, before IPTG induction; line 2, after 0.1 mM IPTG induction at 16°C for 24 h; line 3, supernatant after IPTG induction; line 4, precipitate after IPTG induction; line 5, penetrating liquid; lines 6 to 13/14, 250 mM imidazole-elution product. Notably, MXAN_4468 and the two mutant proteins spontaneously formed either dimers or tetramers that were resistant to SDS treatment in PAGE. (E) Preparation and purification of HrcA with an MBP or a His tag at the C terminus presented by SDS-PAGE. (Left) Line 1, before IPTG induction; line 2, after 0.1 mM IPTG induction at 16°C for 24 h; line 3, supernatant after IPTG induction; line 4, precipitate after IPTG induction; line 5, penetrating liquid; lines 6 to 14, 10 mM maltose-elution product. M, marker: PageRuler™ prestained protein ladder. (Right) Line 1, before IPTG induction; line 2, supernatant after IPTG induction; line 3, precipitate after IPTG induction. M, marker: unstained protein molecular weight (MW) marker (SM0431). Line 4, penetrating liquid; line 5, precipitate dissolved by 1× Tris-EDTA (TE) buffer; line 6, penetrating liquid; lines 7 to 10, 50 mM imidazole-elution product. Download FIG S2, TIF file, 2.9 MB. Copyright © 2022 Zhuo et al. 2022 Zhuo et al. https://creativecommons.org/licenses/by/4.0/ This content is distributed under the terms of the Creative Commons Attribution 4.0 International license.

To explore the phosphorylation and its functional effects, we mutated the *MXAN_4468* gene by altering the 61^st^ aspartic acid to valine and phenylalanine in the DK1622 genome according to references 22 and 23), producing the Δ*4468*::*4468*-D61V and Δ*4468*::*4468*-D61F mutant strains, respectively. This amino acid swapping led to almost the same transcriptional levels of *groES* and *groEL*s as those in the *MXAN_4468* knockout mutant under either normal temperature or heat shock conditions (Fig. 2B and C). To facilitate the phosphorylation check, we further linked a 6×His-encoding sequence to the C terminus of *MXAN_4468* in the wild-type DK1622 and the two mutants. The Phos-tag PAGE analysis showed that only purified MXAN_4468 protein with no addition of HK was not phosphorylated (Fig. 2D, lane 1). However, the MXAN_4468 protein in the supernatant of DK1622, which contains HKs, was phosphorylated (lane 2), but both the the 61^st^ mutant proteins in mutant strains, which also contain HKs, were not (lanes 3 and 4) (Fig. 2D). The results indicate that the 61^st^ aspartic acid is the phosphorylation site of MXAN_4468, and phosphorylation of this amino acid is essential for MXAN_4468 to regulate the transcription of *groES* and *groEL*s.

We performed a pull-down assay of the MXAN_4468 wild-type protein, as well as the D61V and D61F mutant proteins, with the supernatant of disrupted DK1622 cells to screen its potential interacting proteins. The expression and purification of these proteins are shown in Fig. S2D. We noticed that the proteins specifically bound by MBP-MXAN_4468 included two CheA proteins, MXAN_4758 and MXAN_6964, which, however, were not among the binding proteins of the two MXAN_4468 mutants (Table S2). CheA proteins are HKs that are responsible for transferring the phosphoryl group to the RRs in the corresponding TCSs (24, 25). MXAN_4468 is an orphan RR, and the two CheA proteins may be the noncognate HKs for the phosphorylation of MXAN_4468. Interestingly, the *MXAN_4758* and *MXAN_6964* genes belong to the Che8 and Che7 chemosensory pathways, respectively (21). The Che7 system has been reported to be associated with a HEAT repeat domain-containing protein and required for the appropriate coupling of aggregation and sporulation, while the functions of the Che8 system have not yet been identified (21, 26). We knocked out each of the two *cheA* genes from the DK1622 strain. The two deletions led to similar transcriptional levels of *groESL* genes as that resulting from *MXAN_4468* deletion (Fig. 2E). Comparably, the *MXAN_4758* (*cheA8*) deletion mutant had a closer transcriptional level of *groESL* to that of the *MXAN_4468* deletion mutant than the *MXAN_6964* (*cheA7*) deletion mutant. Notably, in the eight chemosensory pathways (21), while five CheA proteins are hybrid HKs (CheA-RR fusion protein) responsible for autophosphorylation, three are typical HKs (single-domain CheA), including the above-mentioned two CheAs and MXAN_6692. The latter is in the Dif chemosensory pathway and was not bound by MXAN_4468 in the pull-down assay. We similarly deleted the *MXAN_6692* gene, which, however, did not change the transcription of *groESL*s (Fig. 2E). The above-described results strongly suggest that *MXAN_4758* and *MXAN_6964* are both responsible for the phosphorylation of MXAN_4468 protein and that *MXAN_4758* is probably more important for this process.

10.1128/mSystems.01056-21.8 TABLE S2 Proteins potentially interacting with MXAN_4468, MXAN_4468-D61V, and MXAN_4468-D61F in M. xanthus DK1622. The proteins interacting with MXAN_4468 that were absent in the results of D61V or D61F are in the colored background. Download Table S2, DOCX file, 0.04 MB. Copyright © 2022 Zhuo et al. 2022 Zhuo et al. https://creativecommons.org/licenses/by/4.0/ This content is distributed under the terms of the Creative Commons Attribution 4.0 International license.

### Phosphorylated MXAN_4468 binds to HrcA to inhibit *groESL* transcription.

HrcA (MXAN_6726) is a negative transcription regulator of duplicate *groEL*s (15). This protein was also pulled down by MXAN_4468 but was not by the two MXAN_4468 mutants. Compared to the Δ*hrcA* mutant, the Δ*4468* mutant showed almost unchanged *groES* and *groEL1* transcriptional levels, but its *groEL2* transcriptional level was approximately three times higher (*t* test, *groEL2*, *P* value < 0.001). Further deletion of *hrcA* in the Δ*4468* mutant caused no more changes in the *groES* and *groEL*s transcriptional levels (Fig. 2F; *t* test, *groES*, *P* value = 0.122; *groEL1*, *P* value = 0.141; *groEL2*, *P* value = 0.088). Thus, compared to the double deletion mutant of *MXAN_4468* and *hrcA*, only *hrcA* without *MXAN_4468* (Δ*4468*) had almost no regulatory effect on the transcription of *groES* and *groEL*s, only *MXAN_4468* without *hrcA* (Δ*hrcA*) negatively and specifically regulated the transcription of *groEL2*, and the presence of both *hrcA* and *MXAN_4468* (DK1622) led to negative regulation of the transcription of *groES* and *groEL*s. The results suggest that MXAN_4468 not only cooperates with HrcA on the transcriptional regulation of *groES* and *groEL*s but also alone specifically inhibits the transcription of *groEL2*.

To investigate the binding ability between MXAN_4468 and HrcA, we constructed two recombinant expression vectors, *4468*-pET32a and *hrcA*-pMAL-c5x, and labeled the C termini of MXAN_4468 and HrcA with His and maltose binding protein (MBP) tags, respectively (the purification of HrcA protein is shown in Fig. S2E). *In vitro* pull-down experiments showed that although there existed some excess proteins in penetrating liquid (Fig. 2G, lane 3), 4468-His and HrcA-MBP were both absorbed on beads for His proteins in the elution of imidazole washing buffer (lane 4), while MBP, mixed with 4468-His, was not absorbed on beads (lane 2). This result further confirmed the presence of a binding interaction between the MXAN_4468 and HrcA proteins.

Through electrophoretic mobility shift assay (EMSA) and isothermal titration calorimetry (ITC) binding experiments, we previously proved that HrcA has the capacity to bind CIRCE1*_groESL1_* or CIRCE*_groEL2_* but no capacity to interact with CIRCE2*_groESL1_* in M. xanthus DK1622 (15). To investigate whether MXAN_4468 affected the binding reaction of HrcA and CIRCE, we added MXAN_4468 and its mutants of D61V and D61F to the reaction mixtures. As shown in Fig. 2H, HrcA alone formed a weak blocking band with CIRCE1*_groESL1_* or CIRCE*_groEL2_*, and the addition of MXAN_4468, but not the MXAN_4468 mutants, significantly enhanced the brightness of the blocking band and completely weakened the brightness of DNA band (lanes 7 and 8); MXAN_4468 itself was unable to bind to the CIRCE elements (lanes 9 and 10). The weak binding ability of HrcA alone to CIRCE sequences suggests its regulatory effect on the transcription of *groES* and *groEL*s, which, however, is inconsistent with the phenotypic comparison results of the Δ*4468* and Δ*4468* Δ*hrcA* mutants (Fig. 2F). In M. xanthus DK1622, CIRCE1*_groESL1_* and CIRCE*_groEL2_* sequences overlap the transcription start site (TSS) and −35 regions of the promoters of *groEL1* and *groEL2*, respectively, and these regions are the binding targets of many essential factors for transcription, such as σ^32^ and HrcA regulators (15). We suggest that the binding of MXAN_4468 to HrcA is necessary for the regulatory role of HrcA in the transcription of *groES* and *groEL*s, probably by enhancing the binding competition ability of HrcA to the CIRCE sequences.

### Phosphorylated MXAN_4468 binds to its own gene sequence to specifically inhibit the transcription of the downstream *groEL2*.

**FIG 3:**
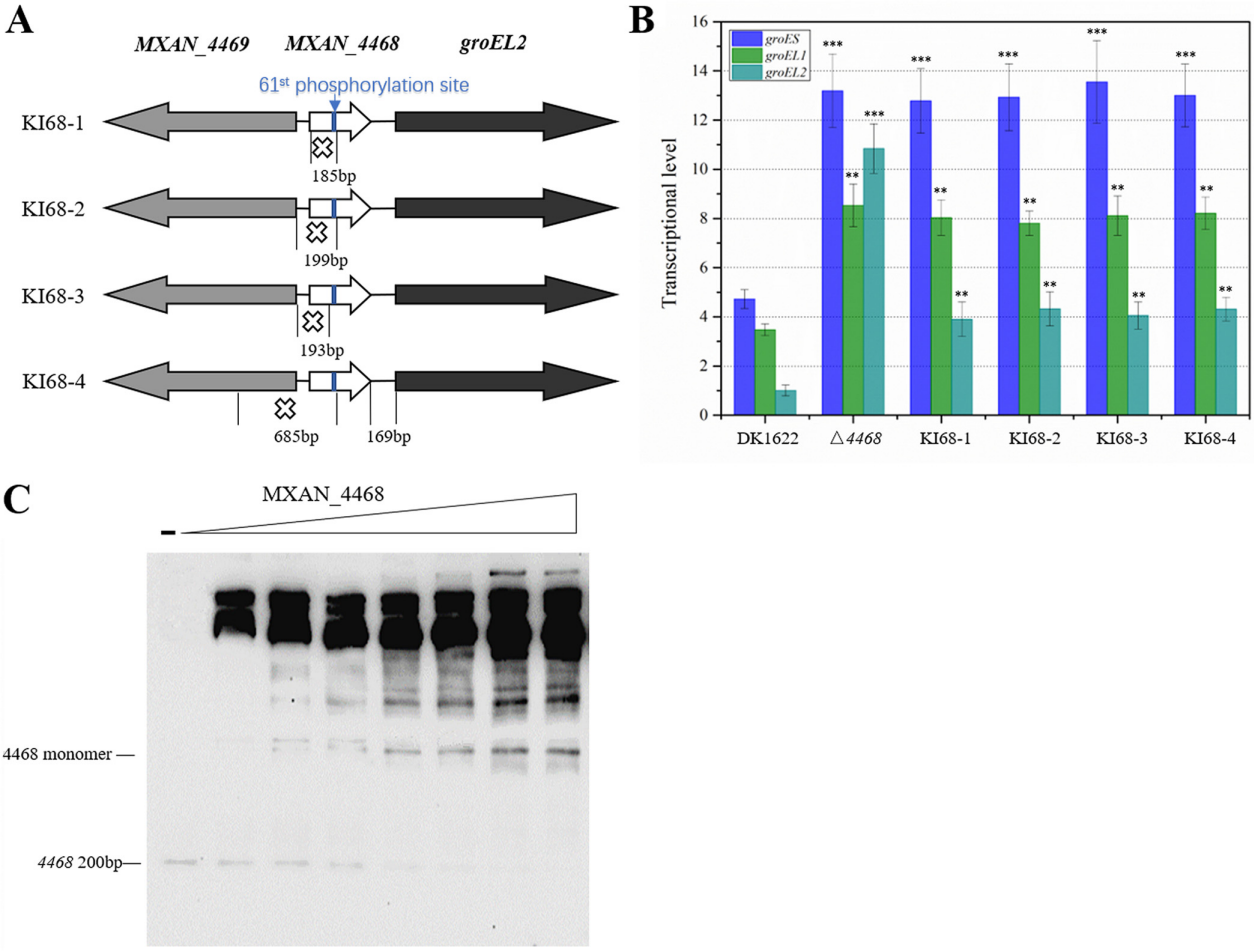
The transcriptional relationship between *MXAN_4468* and its own coding region. (A) Representations of the *MXAN_4468* knockout mutants. (B) qPCR analysis of the transcriptional levels of *groES* and *groEL*s in *MXAN_4468* knockout mutants and the wild-type strain DK1622. The transcriptional level of *groEL2* in DK1622 was set to 1. For statistical analysis, “***” and “**” mean *P* values of <0.001 and <0.01, respectively. (C) Analysis of binding activity between the MXAN_4468 protein and the inner 200-bp 3′-end sequence of *MXAN_4468* using EMSA (presented by native PAGE). The monomers of the MXAN_4468 protein are marked in the diagram. Other protein bands with high concentrations were proven to be the polymers of MXAN_4468 (Fig. S3B to D).

The interval sequence between *MXAN_4468* and *groEL2* is 169 bp long and contains the promoter region of *groEL2*. Because *MXAN_4468* deletion had an additional specific effect on the transcription of *groEL2*, we inferred that the deletion fragment of the Δ*4468* mutant probably contained a regulatory sequence to which MXAN_4468 binds to negatively and specifically regulate the transcription of *groEL2*. To investigate whether there is such a regulatory sequence, we further constructed four mutants at different positions to retain the 3′ end of *MXAN_4468* (Fig. 3A). Briefly, while the 3′-terminal regions were retained in the four partial mutants, the deleted regions were different: in KI68-1, only the 5′-terminal region of *MXAN_4468* was deleted; in KI68-2 and KI68-3, the deleted regions included the promoter region and different 5′-terminal regions (retaining the 61^st^ phosphorylation site or not) of *MXAN_4468*; and in KI68-4, the deletion included the 3′ terminus of the upstream *MXAN_4469*, the promoter region, and a 5′-terminal region of *MXAN_4468*. We did not additionally express the *MXAN_4468* gene in other places of the genome. We found that the transcriptional levels of *groES* and *groEL1* in these four incomplete deletion mutants of *MXAN_4468* were the same as those in the complete deletion mutant, but the transcriptional level of *groEL2* was much lower (Fig. 3B) and was almost the same as that in the Δ*hrcA* mutant (refer to Fig. 2F). Compared to the double deletion mutant of *MXAN_4468* and *hrcA*, the existence of the 3′-end region of *MXAN_4468* and complete *hrcA* (KI68 mutants) had a more negative regulatory effect on the *groEL*2 transcription, similar to that in the mutant with *MXAN_4468* but not *hrcA* (Δ*hrcA*). In other words, the 3′-end region of *MXAN_4468* would be essential to maintain the lower expression of *groEL2*, regardless of the existence of *hrcA*. The results indicate that the 3′-end region of *MXAN_4468*, which was retained in the *MXAN_4468*-incompletely deleted mutants, contains a sequence that is for the specific negative regulation of *groEL2* transcription.

10.1128/mSystems.01056-21.4 FIG S3 (A) Analysis of binding activity between the phosphorylated MXAN_4468 protein and the upstream 370-bp sequence of *groEL2* using EMSA (native PAGE). Base peaks of monomer (C) and polymer (D) bands are shown in EMSA diagrams with purified MXAN_4468 protein (B) as a control. The identification results in panels C and D both were Q1D3Y4 (protein identifier [ID] of MXAN_4468). Download FIG S3, TIF file, 2.2 MB. Copyright © 2022 Zhuo et al. 2022 Zhuo et al. https://creativecommons.org/licenses/by/4.0/ This content is distributed under the terms of the Creative Commons Attribution 4.0 International license.

To confirm this unusual regulatory mechanism, we performed an EMSA of the MXAN_4468 proteins with the upstream 370-bp sequence of *groEL2* (Fig. S3A) or the inner 200-bp 3′-end sequence of *MXAN_4468* (Fig. 3C). The results showed that there were obvious blocking bands between the MXAN_4468 protein and the two DNA sequences, and the brightness of the DNA-protein complexes was enhanced with an increase in the concentration of MXAN_4468 protein. These blocking bands were determined by mass spectrometry to be MXAN_4468 (Fig. S3B to D) and were presumed to be the monomeric and polymeric forms of MXAN_4468. The band intensity showed that the MXAN_4468 proteins preferred to bind to their own DNA sequence in polymeric forms. Besides MXAN_4468, there exist other 19 single-domain RR homologues in M. xanthus DK1622 (Table S1), which were probably able to bind to the 3′-end region of *MXAN_4468* to decrease the *groEL2* transcription. Compared to the *MXAN_4468* complete knockout mutant, the four incomplete deletion mutants (KI68 mutants) had lower *groEL2* transcription (Fig. 3B). In addition, the mutated MXAN_4468 proteins were observed in the Δ*4468*::*4468*-D61V and Δ*4468*::*4468*-D61F strains (Lanes 3 and 4 in Fig. 2D), i.e., the *MXAN_4468* gene was expressed and still maintained the 3′-end region, but the two mutant strains exhibited almost the same *groES* and *groEL*s transcriptional levels as those exhibited by the Δ*4468* mutant, especially for *groEL2* transcription. Due to the distance advantage of the *MXAN_4468* gene, nonphosphorylated MXAN_4468 proteins could combine with the 3′-end region of *MXAN_4468*, thus preventing the binding of other MXAN_4468 homologues on the location. However, the interaction would not lead to negative regulation on the *groEL2* transcription because of nonphosphorylation of the mutated MXAN_4468 proteins. We conclude that the phosphorylated MXAN_4468 protein specifically inhibits the transcription of *groEL2* by binding to its own sequence, thus interfering with the binding of transcriptional factors to the promoter of the downstream *groEL2* gene.

### Global effects of *MXAN_4468* deletion in M. xanthus DK1622 cells.

Our above-mentioned results demonstrated that the phosphorylated MXAN_4468 protein plays a critical role in regulation of the expression of *groES* and *groEL*s, leading to their differential transcriptional levels. GroEL is a type I chaperonin and an essential component in different bacterial species (27, 28). It is required for correct *in vivo* folding of more than 10% of the total proteins in E. coli (10). Our previous studies indicate that the protein clients of the M. xanthus GroELs, although consistent with those of the single E. coli GroEL in their secondary structural features, vary significantly in the substrate spectra, and GroEL1 and GroEL2 have their own exclusive protein clients in addition to the shared clients (13). Moreover, the proteins that were specifically pulled down by MXAN_4468 included not only the CheA and HrcA proteins but also some other regulatory proteins, such as an RNA polymerase sigma 70 factor (MXAN_7454), a transcriptional regulator (MXAN_1757), a sigma 54 transcriptional regulator (MXAN_0907), which are related to stress and transcriptional regulation (Table S2) (the energies for binding of these three proteins to MXAN_4468 were estimated by the JSmol website to be −28.47, −21.32, and −10.97, respectively). These results suggested that MXAN_4468 probably plays a global transcriptional regulator role, not only via the control of transcriptional levels of *groESL*s but also via other regulatory proteins. To explore potential global effects, we performed a transcriptome analysis on the *MXAN_4468* knockout strain and the wild-type strain DK1622 using cells grown for 24 h and 36 h under the normal temperature or heat shock condition. We BLAST searched the reads of transcriptome data against the reference genome of DK1622 using the Bowtie2 program (29) and found that the total mapped value of all samples exceeded 99% (Table S3), which indicates that the samples were not contaminated and that the transcriptome data were reliable.

10.1128/mSystems.01056-21.9 TABLE S3 Comparison of the reads of transcriptomic data and reference genomes. Download Table S3, DOCX file, 0.03 MB. Copyright © 2022 Zhuo et al. 2022 Zhuo et al. https://creativecommons.org/licenses/by/4.0/ This content is distributed under the terms of the Creative Commons Attribution 4.0 International license.

**FIG 4:**
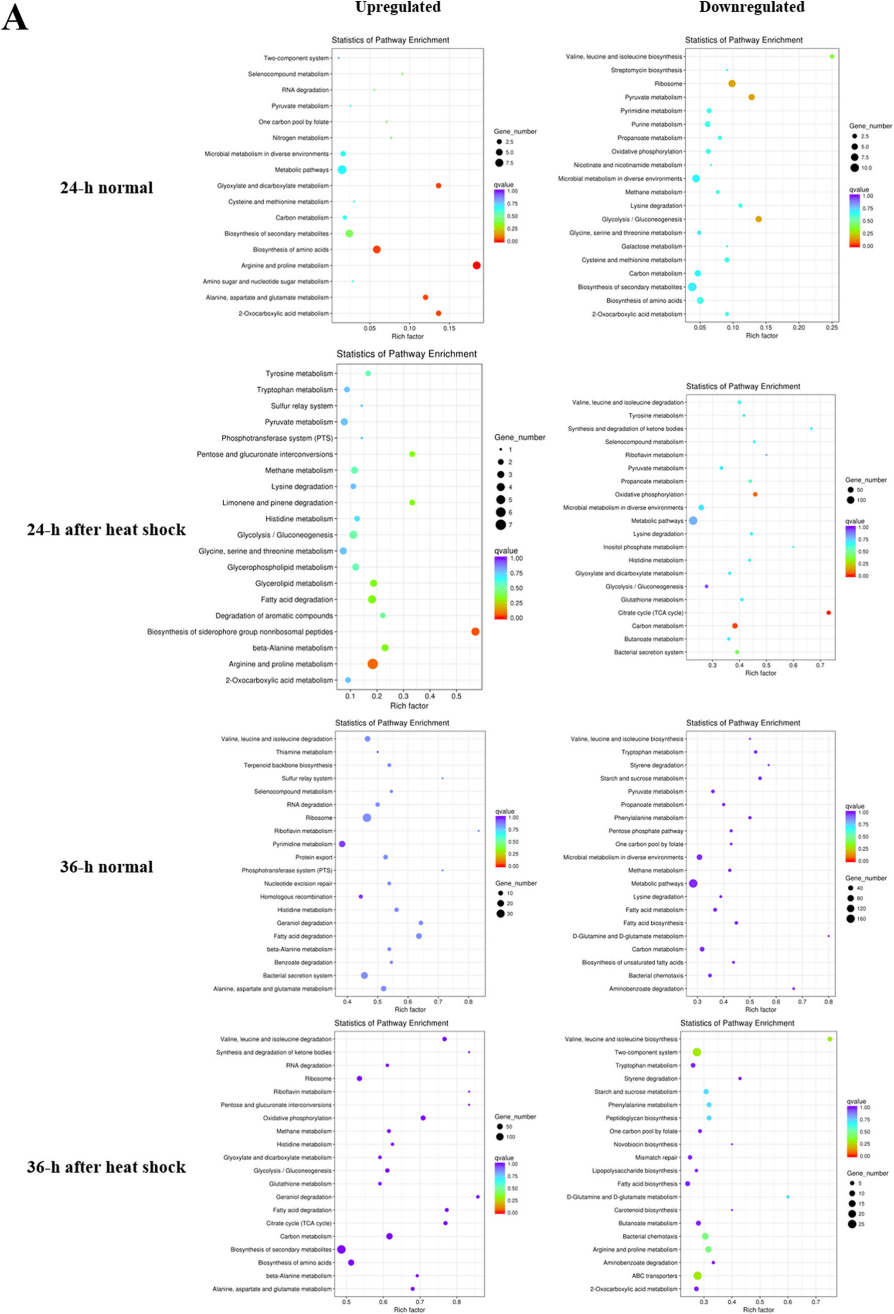

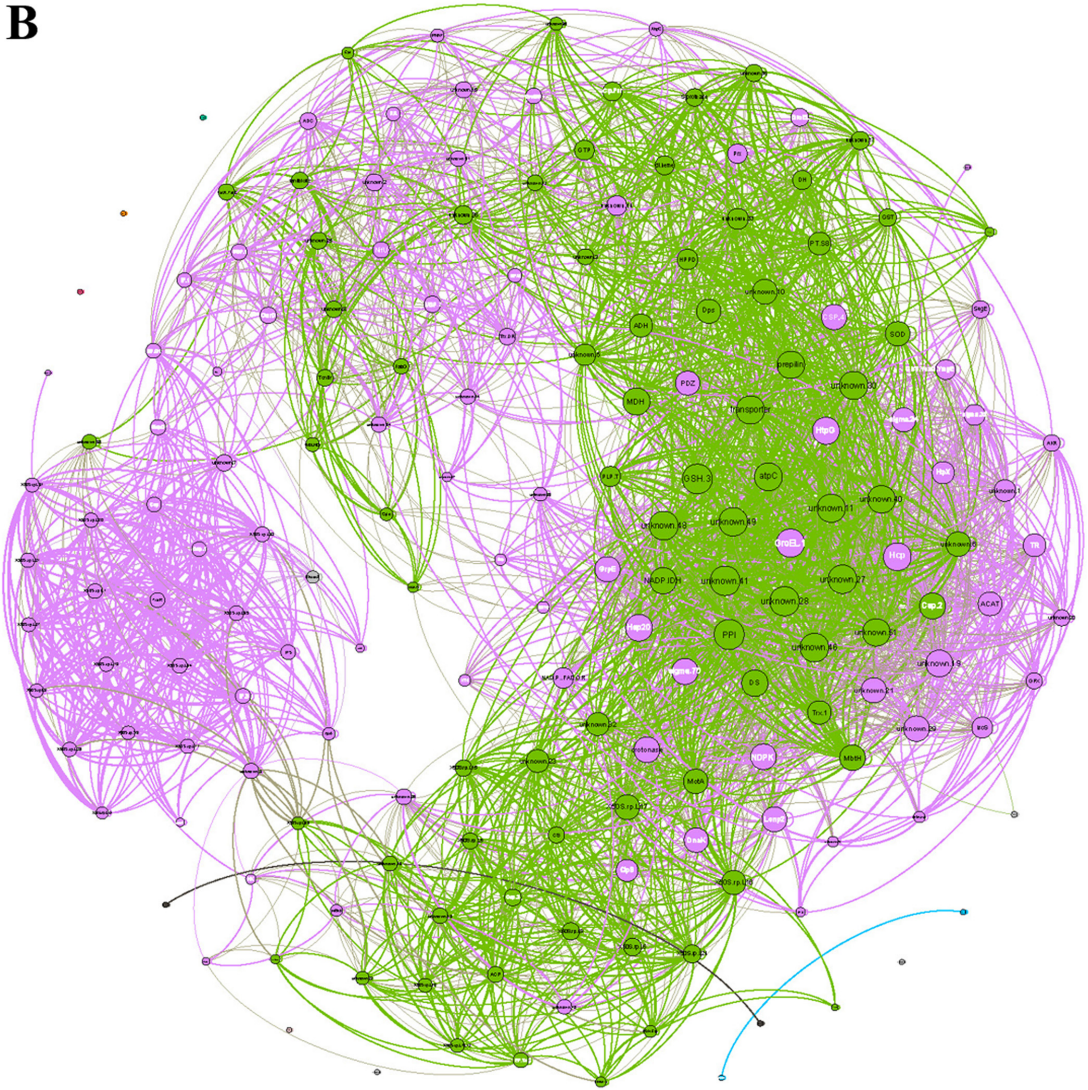
Transcriptomic analysis of the *MXAN_4468* knockout mutants and the wild-type strain DK1622. (A) KEGG enrichment analysis of differentially expressed genes under different conditions. (B) Network analysis of the top 200 differentially expressed genes with high expression in all transcriptomic samples.

We found that the transcriptomes of the Δ*MXAN_4468* strain and DK1622 had significant differences (Fig. S4; for details, see Data Set S1 [sheets 1 to 4]). Functional annotation of the differentially expressed genes in the cells grown for 24 h under the normal temperature condition indicated that the genes that were significantly upregulated by *MXAN_4468* deletion were mainly related to the two-component system and metabolism, while those that were significantly downregulated were mainly related to ribosomes and binding proteins (Fig. 4A). After heat shock, the most significantly upregulated genes were involved in the pathways of metabolism and phosphotransferase system, and the most significantly downregulated genes were related to partial amino acid degradation and oxidative phosphorylation. Under the 36-h normal temperature and heat shock conditions, the significantly upregulated genes were related to amino acid and RNA degradation, and the significantly downregulated genes were related to metabolism and amino acid biosynthesis.

10.1128/mSystems.01056-21.1 DATA SET S1 Differentially expressed genes between the *MXAN_4468* deletion mutant and wild-type DK1622 under different temperature conditions (sheets 1 to 4). Details of the four classes of top 200 differentially expressed genes with high expression are also shown (sheets 5 to 8). Download Data Set S1, XLSX file, 0.5 MB. Copyright © 2022 Zhuo et al. 2022 Zhuo et al. https://creativecommons.org/licenses/by/4.0/ This content is distributed under the terms of the Creative Commons Attribution 4.0 International license.

10.1128/mSystems.01056-21.5 FIG S4 Volcano maps of differentially expressed genes under different conditions. Adjusted P value (*P*_adj_) < 0.05. hs, heat shock. The numbers of upregulated (green) and downregulated (red) genes due to the deletion of *MXAN_4468* are included. For details, refer to Data Set S1 (sheets 1 to 4). Download FIG S4, TIF file, 0.7 MB. Copyright © 2022 Zhuo et al. 2022 Zhuo et al. https://creativecommons.org/licenses/by/4.0/ This content is distributed under the terms of the Creative Commons Attribution 4.0 International license.

To highlight the transcriptional impacts of the *MXAN_4468* deletion, we performed a network analysis of the top 200 differentially expressed genes in all transcriptome samples. These top 200 genes were classified into four classes according to their annotation: stress regulatory proteins (40), metabolites (66), ribosomal proteins (39), and unknown proteins (55) (Data Set S1 [sheets 5 to 8]). Notably, the genes encoding stress regulatory proteins were mostly upregulated (36 of 40; the gene names in white with a purple background in Fig. 4B), while the genes encoding metabolites and ribosomal proteins were either upregulated or downregulated (colored in purple or green). These results suggest that the transcription of the genes encoding stress regulatory proteins was regulated in a similar manner by *MXAN_4468* deletion; i.e., the orphan RR probably played a global negative regulatory role in the transcription of these genes.

### *MXAN_4468* deletion decreases cell competition ability in M. xanthus.

**FIG 5:**
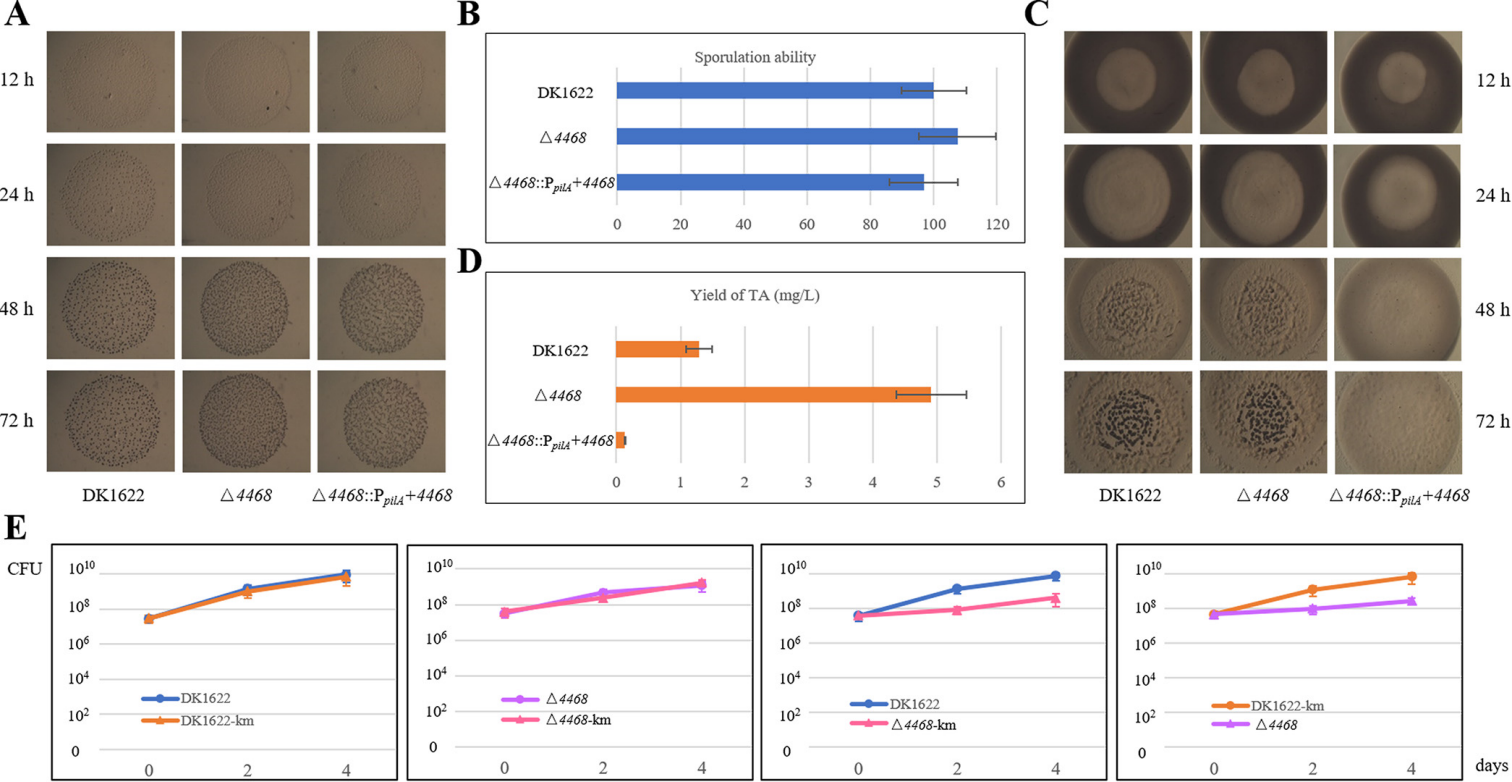
Growth competition and social behavior analyses of the *MXAN_4468* mutants and the wild-type strain DK1622. The development (A), sporulation ability (B), predation (C), and myxovirescin (TA) fermentation level (D) analysis of the *MXAN_4468* knockout and overexpression mutants and the wild-type strain DK1622 are shown. The sporulation ability of DK1622 was set to 100. (E) Coincubation assays of the kanamycin-resistant strain DK1622-km (Δ*4468-*km) and the kanamycin-sensitive strain DK1622 (Δ*4468*). The strains were mixed in a 1:1 ratio and cocultured for 0, 2, and 4 days.

M. xanthus cells have complex social behavior (30) and the ability to produce diverse secondary metabolites (31). We previously determined that the two *groEL* genes have divergent roles in development, predation, and biosynthesis of secondary metabolites (12–14). To efficiently achieve their cellular functions, the expression level of *groEL1* is about four times higher than that of *groEL2*, and their total expression level is approximately equal to the expression level of *groES*, the cochaperone gene of the duplicate *groEL*s (11). However, deletion of the *MXAN_4468* gene led to not only high expressions of the duplicate *groEL* genes but also unbalanced transcription of *groES* and *groEL*s. We found that the *MXAN_4468* deletion had almost no effects on development, sporulation, and predation abilities of M. xanthus (Fig. 5A to C) but markedly increased the biosynthesis of myxovirescin (Fig. 5D). In contrast, the *in situ* overexpression of *MXAN_4468*, which had almost no effect on the transcription of *groES* and *groEL1* but significantly decreased the transcription of *groEL2* (Fig. 1D), had no effects on development and sporulation but caused a weak deficiency in predation and led to near-undetectable myxovirescin biosynthesis. Thus, overexpression of *groEL1* or *groEL2* allowed or improved their related cellular functions but caused no effect on their unrelated functions; i.e., *groEL1* plays an essential role in fruiting body development and sporulation, while *groEL2* is required in predation and myxovirescin biosynthesis (12–14).

Although the high and unbalanced transcription of *groES* and *groEL*s had no significant effects on social behavior, it should be a metabolic burden to the host (32–34) and probably led to weaker competitive ability than that exhibited by the wild-type strain. To assay their growth competitive abilities, we constructed kanamycin-resistant strains of M. xanthus DK1622 and the Δ*4468* mutant and cocultured wild-type and mutant strains to form a competitive environment following the previous protocol (35). When the kanamycin-resistant and -sensitive DK1622 or Δ*4468* strains were cocultured on a casitone-based rich-nutrient (CTT) plate for 0, 2, and 4 days, the two types of cells in the harvested mixtures had the same number of CFU. However, when kanamycin-resistant Δ*4468* and -sensitive DK1622, or kanamycin-resistant DK1622 and -sensitive Δ*4468*, were mixed for cocultivation, the growth of the Δ*4468* mutant was significantly slower than that of the wild-type DK1622 (Fig. 5E). These results indicate that *MXAN_4468* deletion weakened the competitive ability of cells, probably due to the metabolic burden of high and unbalanced transcription of *groES* and *groEL*s.

## DISCUSSION

There are multiple copies in the DK1622 genome of the genes that encode single-domain RR proteins (Table S1). Seven of them are located in different chemosensory pathways (21) and the rest are orphan ones, like *MXAN_4468*. Previous studies have revealed that *MXAN_6693* (*difD*) in the Dif system (the Che2 system) is related to the chemotaxis of lipids (36, 37), *MXAN_2684* and *MXAN_6956*, belonging to the Che4 and Che6 systems, respectively, are both responsible for aggregation and sporulation (38, 39), and *MXAN_6965* in the Che7 system is important for resistance to temperature stress and for the production of viable spores (26). Little is known as yet about the function of the Che8 chemosensory pathway, as well as the substantial orphan RRs. In this study, we found that an orphan RR (MXAN_4468) plays a central role in tuning the transcriptional differences of *groESL*s via a novel functioning mode. First, the RR binds to HrcA and thereby enhances its ability to bind to the CIRCE elements, which enables it to achieve the transcriptional inhibition of the duplicate *groEL*s. Second, the RR binds to the 3′ end of its own gene sequence, thereby specifically decreasing the transcription of the following *groEL2* gene. These functions require phosphorylation of the 61^st^ aspartic acid residue of the RR. In addition to these specific functions, MXAN_4468 is probably involved in global regulation of cellular functions, indicated by significant transcriptomic changes caused by *MXAN_4468* deletion. The genes specifically regulated by MXAN_4468 are the duplicate *groEL*s, whose encoded proteins play essential roles in protein folding, assembly, and transport. In addition, pull-down results showed that MXAN_4468 could bind to some other transcriptional regulators and kinases (Table S2). It is thus unclear whether the transcriptomic changes were caused by MXAN_4468 directly, by GroELs indirectly, or by both.

**FIG 6:**
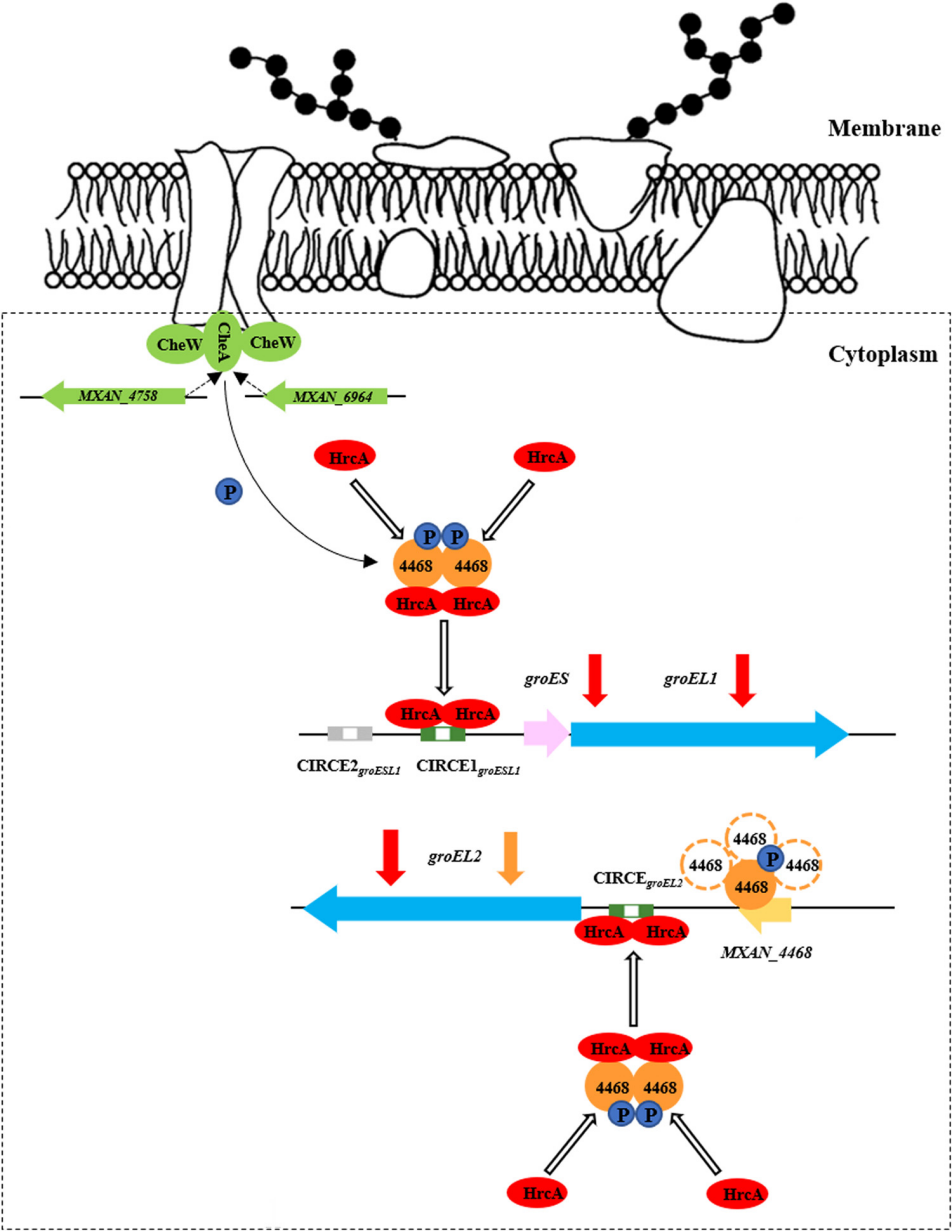
Schematic of the transcriptional regulation of *groES* and *groEL*s by MXAN_4468 in M. xanthus DK1622.

In Bacillus subtilis cells, the HrcA protein is usually present in an inactive monomer form (40, 41), which is dimerized into an active form for binding to the CIRCE elements (42). Structural modeling indicated that dimerized MXAN_4468 is much more stable than dimerized HrcA (the dimerization energy of HrcA is −1.32, but that of the MXAN_4468 dimer is −95.77 [Fig. S5A]; the binding energy between MXAN_4468 and HrcA is −26.73 [Fig. S5B]). We posit that the MXAN_4468 dimer may recruit and bind to HrcA, consequently improving the formation of an active HrcA dimer for binding to the CIRCE elements in the promoter region of the duplicate *groEL*s. We propose a functioning model for the negative regulatory role of MXAN_4468 in the transcription of *groEL*s in M. xanthus cells (Fig. 6). The regulation of *groESL1* by MXAN_4468 via the recruitment of HrcA leads to the formation of a more active dimer to play a negative regulatory role in combination with CIRCE. This protein-protein interaction may promote the binding of this negative regulator to its target sequence, thus further enhancing its regulatory effect. In addition, to regulate *groEL2*, MXAN_4468, in polymeric forms, bound to the internal sequence of its own gene to further inhibit the transcription of *groEL2*. The binding of the MXAN_4468 protein to the *MXAN_4468* 3′-end DNA sequence was analyzed using nucleic acid-protein dock (NPdock) software (43), which showed that the C-terminal region of MXAN_4468 was likely responsible for binding to its own gene sequence on a 12-bp reverse complementary palindromic sequence (Fig. S5C).

10.1128/mSystems.01056-21.6 FIG S5 Modeling analysis of MXAN_4468 self-dimerization (A) and the interaction between the MXAN_4468 (red) and HrcA (blue) proteins (B). (C) DNA-protein complex structure analysis of the MXAN_4468 protein and its own gene sequence. The predicted potential DNA-binding region was a 12-bp reverse complementary palindromic sequence. Download FIG S5, TIF file, 2.7 MB. Copyright © 2022 Zhuo et al. 2022 Zhuo et al. https://creativecommons.org/licenses/by/4.0/ This content is distributed under the terms of the Creative Commons Attribution 4.0 International license.

There are 119 RRs in M. xanthus, and more than 50% of them are orphan. MXAN_4468 is a typical orphan RR; i.e., its function requires phosphorylation but without an HK gene adjacent on the genome (8). Here, we report that two noncognate HKs are the upstream components of MXAN_4468. The two corresponding *cheA* genes, i.e., *MXAN_6964* and *MXAN_4758*, belong to the Che7 and Che8 systems, respectively (21). Many organisms carry a substantial fraction of their two-component genes as orphans probably from duplication or loss of one component, which might be a mechanism for generating cross-regulation systems (44). The cross talk between noncognate HKs and an orphan RR (45) suggests complex and global regulation of MXAN_4468. Our network analysis suggested that among the multiple RR copies in M. xanthus DK1622, the special location of *MXAN_4468* may have extensive effects on the expression of stress regulatory proteins and yet have no correlation with other RR genes involved in the regulation of transcription. The global transcriptional effects of MXAN_4468 may be directly or indirectly related to the functions of *groEL*s. We speculate that in bacteria possessing multiple copies of orphan RR genes, these RR genes are involved in regulating the transcriptional symphony in cells to ensure that complex biological functions can occur.

## MATERIALS AND METHODS

### Cultures, plasmids, and growth conditions.

The strains, plasmids, and primers that were used in this study are shown in Table S4. The M. xanthus strains were cultivated in CTT medium (46) for growth assays. E. coli strains were routinely grown on LB agar or in LB broth. E. coli strains were grown at 37°C, while *Myxococcus* strains were incubated at 30°C. The temperature for heat shock treatment was 42°C. When required, kanamycin, ampicillin, and chloramphenicol were added to the media at final concentrations of 40, 100, and 34 μg/mL, respectively.

10.1128/mSystems.01056-21.10 TABLE S4 Bacterial strains, plasmids, and primers used in this study. Download Table S4, DOCX file, 0.03 MB. Copyright © 2022 Zhuo et al. 2022 Zhuo et al. https://creativecommons.org/licenses/by/4.0/ This content is distributed under the terms of the Creative Commons Attribution 4.0 International license.

### Evolutionary relationship analysis.

The analysis of evolutionary relationships was completed using MEGA6 software (47). Briefly, protein sequences were constructed by the neighbor-joining method (NJ model) and pairwise deleted (pairwise deletion of gaps), and different evolutionary tree models were selected according to the type of protein. All tree self-expansion tests were set at 1,000 repeats to evaluate the stability of the nodes.

### Deletion of *MXAN_4758*, *MXAN_6964*, *MXAN_6692*, *MXAN_4468*, or *MXAN_4468* and *hrcA*.

An in-frame deletion in M. xanthus was performed using the positive-negative KG cassettes described by Ueki et al. (48). Genomic DNA from DK1622 served as the template for the PCR amplification of the upstream and downstream homologous arms using Phanta Super-Fidelity DNA polymerase (Vazyme). The fragments were cloned into SmaI-digested pBJ113 to construct the deletion plasmid pBJ-*4758*, pBJ-*6964*, pBJ-*6692*, or pBJ-*4468*, which was then transferred via electroporation into M. xanthus DK1622 cells. Individual kanamycin-resistant colonies were selected and then inoculated onto CTT agar plates supplemented with 1% galactose for the second round of screening. Deletion mutants were identified by the phenotypes of galactose resistance and kanamycin sensitivity, as well as by PCR verification. The *MXAN_4468* and *hrcA* double-knockout mutant was based on the *MXAN_4468* knockout mutant, in which the *hrcA* gene was deleted.

### Construction of *MXAN_4468 in situ* complement or overexpression mutant.

An *MXAN_4468 in situ* complement or overexpression mutant was constructed on the basis of the obtained *MXAN_4468* knockout mutant, and then a *4468* or P*_pilA_* plus *4468* fragment was inserted *in situ* by the secondary homologous recombination method to construct the *in situ* complement or overexpression mutant.

### qPCR analysis.

M. xanthus DK1622 and mutants were inoculated into CTT medium at an initial concentration of 1 × 10^7^ cells/mL and collected after 24 h of incubation. The cultures were harvested at the testing time points, and the RNA was extracted immediately using a bacterial total RNA isolation kit (Sangon Biotech) according to the manufacturer’s instructions. The purified RNA extracts were reverse transcribed to cDNA. Quantitative real-time PCR was performed with a total reaction volume of 20 μL containing 1 μL of 250 nM primers, 10 μL of SYBR green PCR master mix, 8.5 μL of RNase-free water, and 0.5 μL of a 10-fold-diluted cDNA template. The *gapA* gene was used as the reference. The primers for real-time PCR are listed in Table S4.

### Transcriptome analysis.

Differential expression analysis of M. xanthus DK1622 and mutants subjected to two temperature conditions (normal and heat shock) was performed using the DESeq R package (1.18.0) (49). DESeq provides statistical routines for determining differential expression in digital gene expression data using a model based on the negative binomial distribution. The resulting *P* values were adjusted using Benjamini and Hochberg’s approach (50) for controlling the false-discovery rate. Genes found by DESeq and with an adjusted *P* value of less than 0.05 were assigned as differentially expressed.

The Kyoto Encyclopedia of Genes and Genomes (KEGG) is a database resource for understanding high-level functions and utilities of a biological system, such as a cell, an organism, and an ecosystem, from molecular-level information, especially large-scale molecular data sets generated by genome sequencing and other high-throughput experimental technologies (http://www.genome.jp/kegg/) (51). We used KEGG Orthology-Based Annotation System software to test the statistical enrichment of differentially expressed genes in KEGG pathways.

### Network analysis.

The network was based on the transcriptomic data, such that each node (or vertex) in the network represents a gene (or a protein), and each edge indicates a strong and significant transcriptional correlation. All analyses were performed in R version 3.5.0. The degree of centrality of each node was measured to determine the importance of nodes in the network. Accordingly, all of the nodes were classified into three categories based on the abundance of links with other members in the network: key nodes (vertices with the top 20% centrality), peripheral nodes (vertices with the lowest 20% centrality), and moderate nodes (the remainder of the vertices). All analyses were performed using psych package version 1.8.4 (52).

### Protein structure modeling analysis.

Based on the amino acid sequence of the individual wild-type and mutant protein, the protein structures were modeled using the I-TASSER tool (https://zhanglab.ccmb.med.umich.edu/I-TASSER/) (53). Prediction of protein-protein interactions was performed using the Prism 2.0 tool (http://cosbi.ku.edu.tr/prism/) (54).

### Cell phenotypic analysis.

### (i) Analysis of developmental ability and calculation of sporulation rate.

Briefly, each strain was cultured in CTT/CTT-resistant liquid medium for 20 to 24 h, and then the bacterial concentrations of the growing cultures were adjusted to 5 × 10^9^ cells/mL (optical density at 600 nm [OD_600_] = 35). Subsequently, 10-μL volumes of bacterial solutions were added onto TPM (Tris-HCl phosphate medium) plates, and the plates were incubated at 30°C. The development of fruiting bodies was observed and recorded using a stereoscopic microscope at 12-h intervals. After 5 days of culture, five fruiting bodies of each strain were scraped and cultured in the same centrifugal tube, 100 μL of TPM buffer was added to the tubes, and the bacterial suspensions were subjected to three treatments of low-power ultrasound (<200 W, 4 s) to evenly disperse the cells. The suspensions were then incubated in a bath at 50°C for 2 h. The treated spore suspensions were diluted 10 times with TPM buffer. Subsequently, 50-μL bacterial suspensions of appropriate dilutions were mixed evenly with melted CTT soft agar (0.3% agar) and spread onto CTT/CTT-resistant plates. This process was repeated three times for every strain suspension. The plates were cultured at 30°C for 5 days, and then the number of colonies on the plates was counted and the sporulation rate of each strain was calculated.

### (ii) Analysis of predation ability.

E. coli DH5α was cultured in LB liquid medium for 12 h. Based on the detected OD_600_ value, the bacterial concentration was adjusted to an OD_600_ of 100 using TPM buffer, and 30 μL was inoculated onto a water (WAT) plate (containing only CaCl_2_). The mutants and wild-type strain of *Myxococcus* were cultured in CTT liquid medium for 20 to 24 h. The bacterial concentrations of these growing bacterial cultures were adjusted to 5 × 10^9^ cells/mL (OD_600_ = 35) using TPM buffer, and 2 μL of each bacterial suspension was inoculated onto the middle of an E. coli-inoculated WAT plate. This process was repeated three times for every strain suspension. After drying, the colonies were incubated at 30°C. The predation activity was observed and photographed using a stereoscopic microscope at 12-h intervals.

### Determination of the fermentation level of myxovirescin (TA).

The wild-type DK1622 and mutants were activated on CTT/CTT-resistant solid plates and inoculated into CYE liquid medium for 20 to 24 h. Subsequently, 1% ion-exchange resin was added to each medium, and the culturing was continued for approximately 2 days until the later stage of growth stability. The supernatant was removed to collect the bacteria and resin, and the bacteria were gently washed with double-distilled water (ddH_2_O). Each sample was extracted with approximately 8 mL of methanol and shaken overnight at a uniform speed. This step was repeated two more times. The combined extracts were concentrated to approximately 3 mL using a vacuum concentration dryer. The concentrated solution was centrifuged at 12,000 rpm for 15 min at 4°C, and the supernatant was used for the next step. The production of TA in each strain was detected by HPLC with a *Myxococcus* (TA) standard as a control.

### MXAN_4468 protein purification.

The heterogeneously expressed MXAN_4468 proteins were purified with some modifications. BL21 cells were grown at 37°C in LB medium with 100 μg/mL of ampicillin to an OD_600_ of 0.6 to 0.8 and were induced with 1 mM isopropyl-β-d-thiogalactopyranoside (IPTG). The cells were resuspended in lysis buffer (25 mM Tris-HCl [pH 8.0], 300 mM NaCl, 2 mM dithiothreitol) and sonicated. After gentle ultrasonic treatment, the samples were centrifuged at 12,000 rpm for 30 min at 4°C. The supernatant of His-tagged MXAN_4468 was applied on Ni^2+^-nitrilotriacetic acid (NTA) and then purified by ion-exchange chromatography using a Sephadex 200 gel filtration column. The protein concentration was determined using a microconcentration detector.

### EMSAs.

The purified MXAN_4468 proteins with different concentrations were incubated with DNA at 30°C for 30 min and then subjected to nondenatured polypropylene gel electrophoresis (90 V, 1 h). The gel was visualized by detecting chemiluminescence after blocking it with the solution and antibody (streptavidin-horseradish peroxidase [HRP]) in the electrophoretic mobility shift assay (EMSA) kit (Thermo Fisher).

### Construction of key site mutation expression vector and mutant strains.

The aspartic acid at position 61 (GAC) of MXAN_4468 was mutated to valine (GTC) or phenylalanine (TTC). The expression vectors pET32a, with a His tag, and pMAL-c5X, with an MBP tag, were selected. The mutation-harboring recombinant vector was constructed using the ClonExpress II one-step cloning kit (Vazyme). The same homologous recombination method was used to construct a pBJ113 plasmid containing the *MXAN_4468* gene with a key site mutation, together with its upstream and downstream homologous arms.

### Phos-tag PAGE.

A His tag was attached to the C terminus of MXAN_4468 and the mutant proteins. To ensure correct formation of the spatial structure of the fusion protein, a linker sequence (TCTGGCTCGAGCTCTGGCGCGCCGGGC) was added between the fusion parts. Phos-tag composite electrophoresis adhesive was prepared using the Phos-tag acrylamide AAL-107 kit. When electrophoresis was performed in the Phos-tag composite electrophoresis gel, the specific binding of Phos-tag to the phosphate group of the phosphorylated protein hindered its migration, resulting in lower mobility of the phosphorylated protein than of the unphosphorylated protein (55). During the experiment, a sufficient amount of commercialized histidine protein kinase (l-histidine K1) was added to activate the phosphorylation of MXAN_4468.

### Pull-down analysis for potential binding proteins.

The mixtures of 4468-pMAL, 4468-D61V-pMAL, and 4468-D61F-pMAL were induced and purified, and pMAL (MBP-tagged protein) was established as the negative control. Strain DK1622 cultured on solid medium was transferred to CTT liquid medium and cultured for 20 to 24 h. The cells were collected by centrifugation, washed three times with TPM buffer, then suspended in a heavy suspension buffer, and lysed by ultrasound. After centrifugation, appropriate amounts of supernatant and 4468-MBP plus MBP filler, 4468-D61V-MBP plus filler, 4468-D61F-MBP plus filler, MBP plus filler, or only filler were mixed and incubated at 4°C for 4 h. The incubated mixture was washed through the column with buffer, and then 10 mM maltose was added to elute MXAN_4468 and the protein bound to the filler. The eluted-protein-containing solutions obtained from the experimental and control groups were sent to Shanghai Zhong Ke Xin Sheng Ming Company for identification by mass spectrometry.

### Co-culture of strains.

The nonresistant and resistant strains were cultured in CTT/CTT-km liquid medium at 30°C for 20 to 24 h. The cells were collected, and their concentrations were adjusted to 5 × 10^9^ cells/mL. The nonresistant and resistant strains were mixed at a 1:1 (vol/vol) ratio, and 5-μL volumes of the obtained bacterial solutions were inoculated onto CTT plates. After culturing at 30°C for 0, 2, and 4 days, the whole colonies were scraped and resuspended in 500 μL of TPM buffer. The resulting bacterial suspensions were diluted 10 times, and the suitable four dilutions of three repeated 50-μL suspensions were mixed evenly with 2.5 mL of semisolid CTT (0.3% agar) and then spread onto the CTT/CTT-km plates. After 5 days of culture, the number of colonies on the plate was counted and the CFU of each strain were calculated.
