# Supplementary material for: A Dual-Functional Orphan Response Regulator Negatively Controls the Differential Transcription of Duplicate groELs and Plays a Global Regulatory Role in Myxococcus
Source: mSystems. 2022 Mar 30;7(2):e01056-21. doi: 10.1128/msystems.01056-21 (PMC9040617; doi:10.1128/msystems.01056-21)
Supplement: TABLE S1 [file msystems.01056-21-st001.docx]

**Table S1** Occurrences of cdd388505 protein family encoding genes adjacent to *groEL*s in sequenced myxobacteria and cdd388505 encoding genes in *M. xanthus* DK1622.

cdd388505 encoding genes adjacent to *groEL*s in sequenced myxobacteria

| **Strain** | **Locus_tag** | **Strand** | **Accession** |
| --- | --- | --- | --- |
| ***Archangium gephyra* DSM 2261** | AA314_RS21795 | + | WP_047857047.1 |
|  | AA314_RS21805 | - | WP_047857049.1 |
| ***Corallococcus coralloides* DSM 2259** | COCOR_RS16525 | + | WP_014396127.1 |
|  | COCOR_RS16535 | - | WP_043323215.1 |
| ***Cystobacter fuscus* DSM 2262** | D187_RS38360 | + | WP_043433682.1 |
|  | D187_RS38370 | - | WP_002630379.1 |
| ***Cystobacter violaceus* Cb vi76** | Q664_RS39220 | + | WP_043408217.1 |
|  | Q664_RS39230 | - | WP_043408223.1 |
| ***Hyalangium minutum* DSM 14724** | DB31_RS11640 | + | WP_044186441.1 |
|  | DB31_RS11650 | - | WP_044186444.1 |
| ***Myxococcus stipitatus* DSM 14675** | MYSTI_RS24555 | - | WP_052351173.1 |
| ***Myxococcus xanthus* DK 1622** | MXAN_RS21700 | - | WP_011554466.1 |
| ***Myxococcus macrosporus* HW-1** | LILAB_RS29985 | - | WP_043711631.1 |
| ***Myxococcus fulvus* 124B02** | MFUL124B02_RS27275 | - | WP_046714603.1 |
| ***Myxococcus xanthus* DZ2** | MXDZ_RS0219780 | + | WP_011554466.1 |
| ***Myxococcus xanthus* DZF1** | MXF1_RS0137870 | + | WP_011554466.1 |
| ***Stigmatella aurantiaca* DW4/3-1** | STAUR_RS23740 | + | WP_002615238.1 |
|  | STAUR_RS23750 | - | WP_013376459.1 |
| ***Anaeromyxobacter dehalogenans* 2CP-1** | A2CP1_RS07865 | - | WP_012632846.1 |
| ***Anaeromyxobacter dehalogenans* 2CP-C** | ADEH_RS12375 | + | WP_011421445.1 |
| ***Anaeromyxobacter* sp. K** | ANAEK_RS07375 | - | WP_012525522.1 |

cdd388505 encoding genes in *M. xanthus* DK1622

| **Locus tag** | **Start** | **Stop** | **Strand** | **Length (bp)** | **Protein product** | CDD | **Old locus tag** | **Protein name** | **Chemosensory pathway*** |
| --- | --- | --- | --- | --- | --- | --- | --- | --- | --- |
| MXAN_RS03680 | 871242 | 871607 | - | 366 | WP_011550889.1 | 388505 | MXAN_0763 | response regulator |  |
| MXAN_RS34755 | 8762033 | 8762398 | + | 366 | WP_011557097.1 | 388505 | MXAN_7178 | response regulator |  |
| MXAN_RS32405 | 8234253 | 8234621 | - | 369 | WP_011556618.1 | 388505 | MXAN_6693 | response regulator DifD | 2 |
| MXAN_RS33885 | 8563251 | 8563619 | + | 369 | WP_011556920.1 | 388505 | MXAN_7001 | response regulator |  |
| MXAN_RS34620 | 8731621 | 8731989 | + | 369 | WP_011557069.1 | 388505 | MXAN_7150 | response regulator |  |
| MXAN_RS23125 | 5962519 | 5962893 | - | 375 | WP_011554746.1 | 388505 | MXAN_4759 | response regulator | 8 |
| MXAN_RS29170 | 7442418 | 7442792 | + | 375 | WP_011555961.1 | 388505 | MXAN_6012 | response regulator |  |
| MXAN_RS14345 | 3470329 | 3470706 | + | 378 | WP_011553018.1 | 388505 | MXAN_2962 | response regulator |  |
| MXAN_RS33715 | 8524624 | 8525001 | - | 378 | WP_011556886.1 | 388505 | MXAN_6965 | response regulator | 7 |
| MXAN_RS35640 | 8990119 | 8990496 | - | 378 | WP_020478873.1 | 388505 | MXAN_7364 | response regulator |  |
| MXAN_RS22550 | 5828570 | 5828950 | - | 381 | WP_026113988.1 | 388505 | MXAN_4645 | response regulator |  |
| MXAN_RS23085 | 5954090 | 5954470 | - | 381 | WP_011554738.1 | 388505 | MXAN_4751 | response regulator | 8 |
| MXAN_RS02260 | 525475 | 525858 | + | 384 | WP_011550595.1 | 388505 | MXAN_0462 | response regulator |  |
| MXAN_RS13000 | 3132171 | 3132554 | + | 384 | WP_011552752.1 | 388505 | MXAN_2684 | response regulator | 4 |
| MXAN_RS27570 | 7051515 | 7051898 | + | 384 | WP_002634498.1 | 388505 | MXAN_5688 | response regulator |  |
| MXAN_RS33730 | 8528467 | 8528850 | + | 384 | WP_020477982.1 | 388505 | MXAN_6968 | response regulator |  |
| MXAN_RS21700 | 5532294 | 5532680 | - | 387 | WP_011554466.1 | 388505 | MXAN_4468 | response regulator |  |
| MXAN_RS29270 | 7465215 | 7465601 | - | 387 | WP_011555982.1 | 388505 | MXAN_6033 | response regulator | 5 |
| MXAN_RS33670 | 8512629 | 8513015 | - | 387 | WP_011556877.1 | 388505 | MXAN_6956 | response regulator |  |
| MXAN_RS20645 | 5214781 | 5215176 | + | 396 | WP_011554256.1 | 388505 | MXAN_4253 | response regulator |  |

* Bacterial chemotaxis systems were classified into 8 pathways due to their different comprises and conserved domains. The chemosensory pathways that RRs are involved in are obtained from reference 21.
