# Supplementary figures and images for: A Dual-Functional Orphan Response Regulator Negatively Controls the Differential Transcription of Duplicate groELs and Plays a Global Regulatory Role in Myxococcus
Source: mSystems. 2022 Mar 30;7(2):e01056-21. doi: 10.1128/msystems.01056-21 (PMC9040617; doi:10.1128/msystems.01056-21)

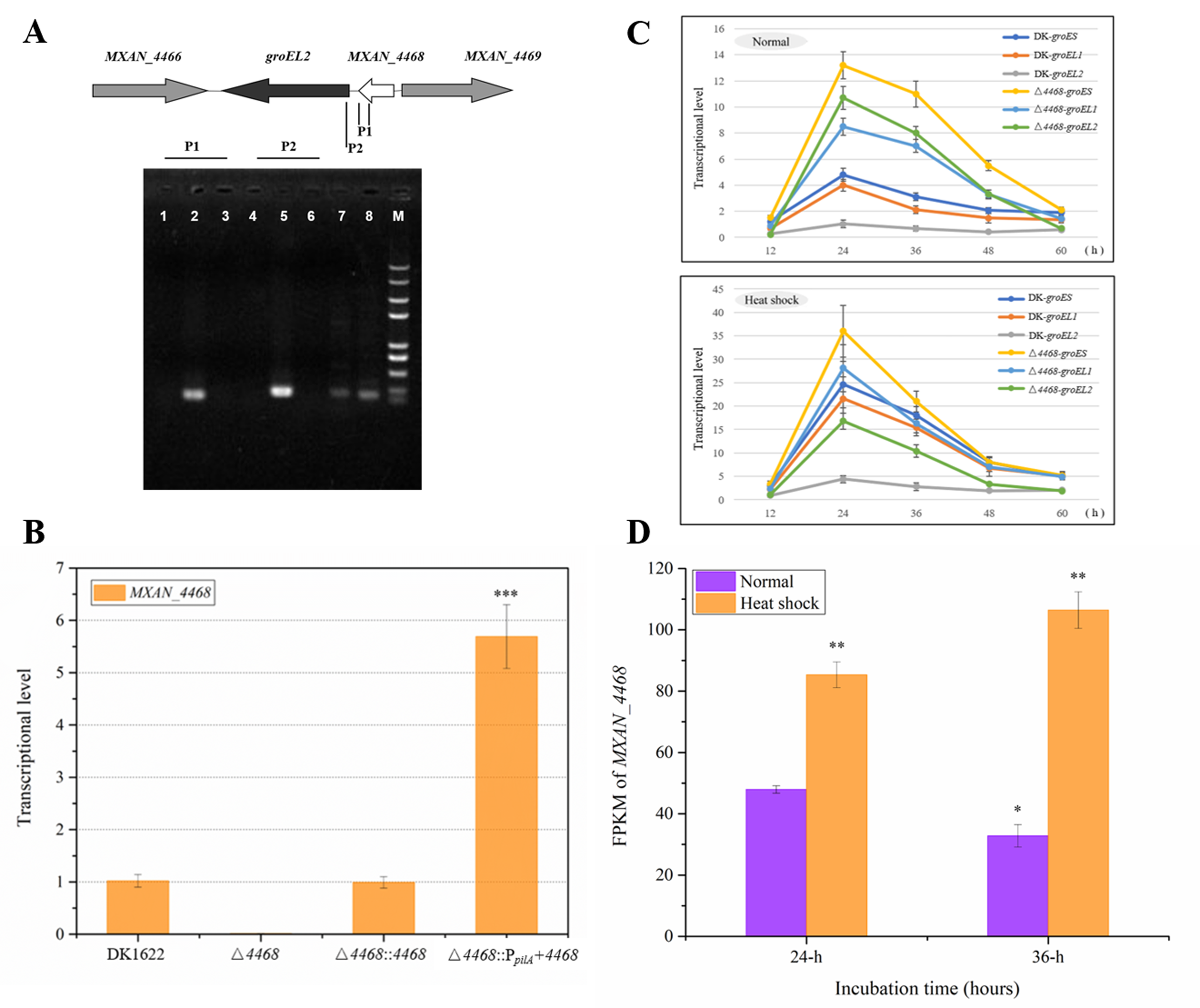

Supplement: FIG S1 [file msystems.01056-21-sf001.tif]

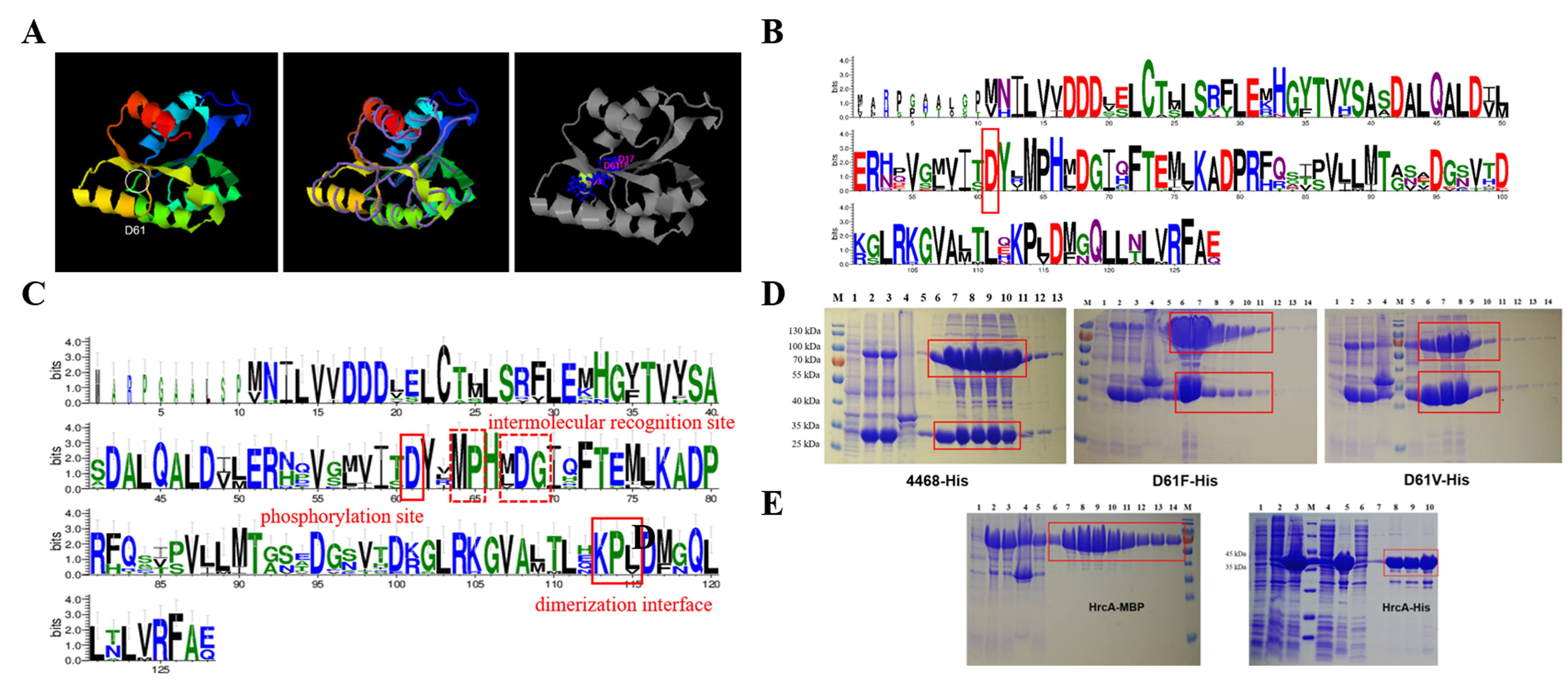

Supplement: FIG S2 [file msystems.01056-21-sf002.tif]

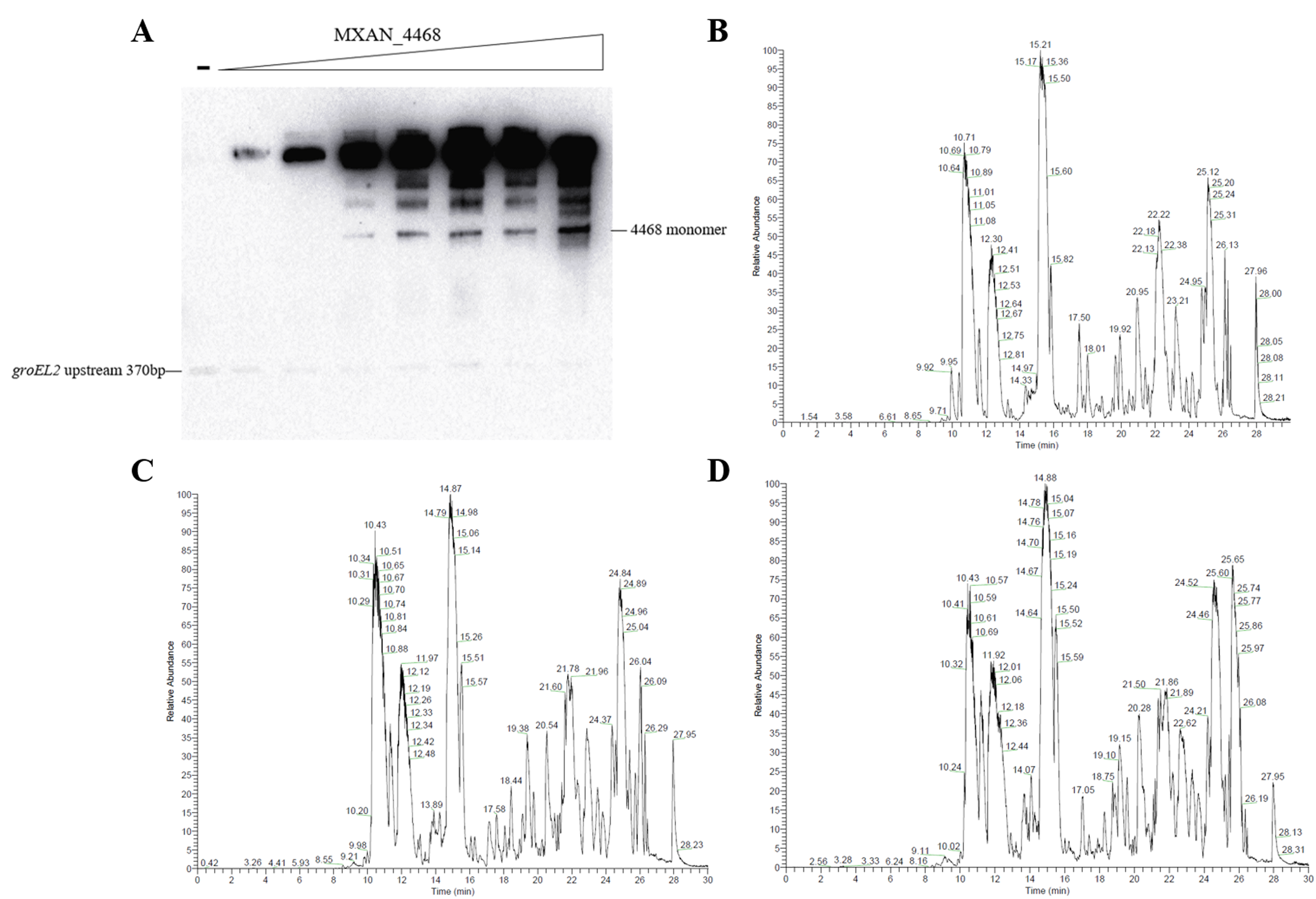

Supplement: FIG S3 [file msystems.01056-21-sf003.tif]

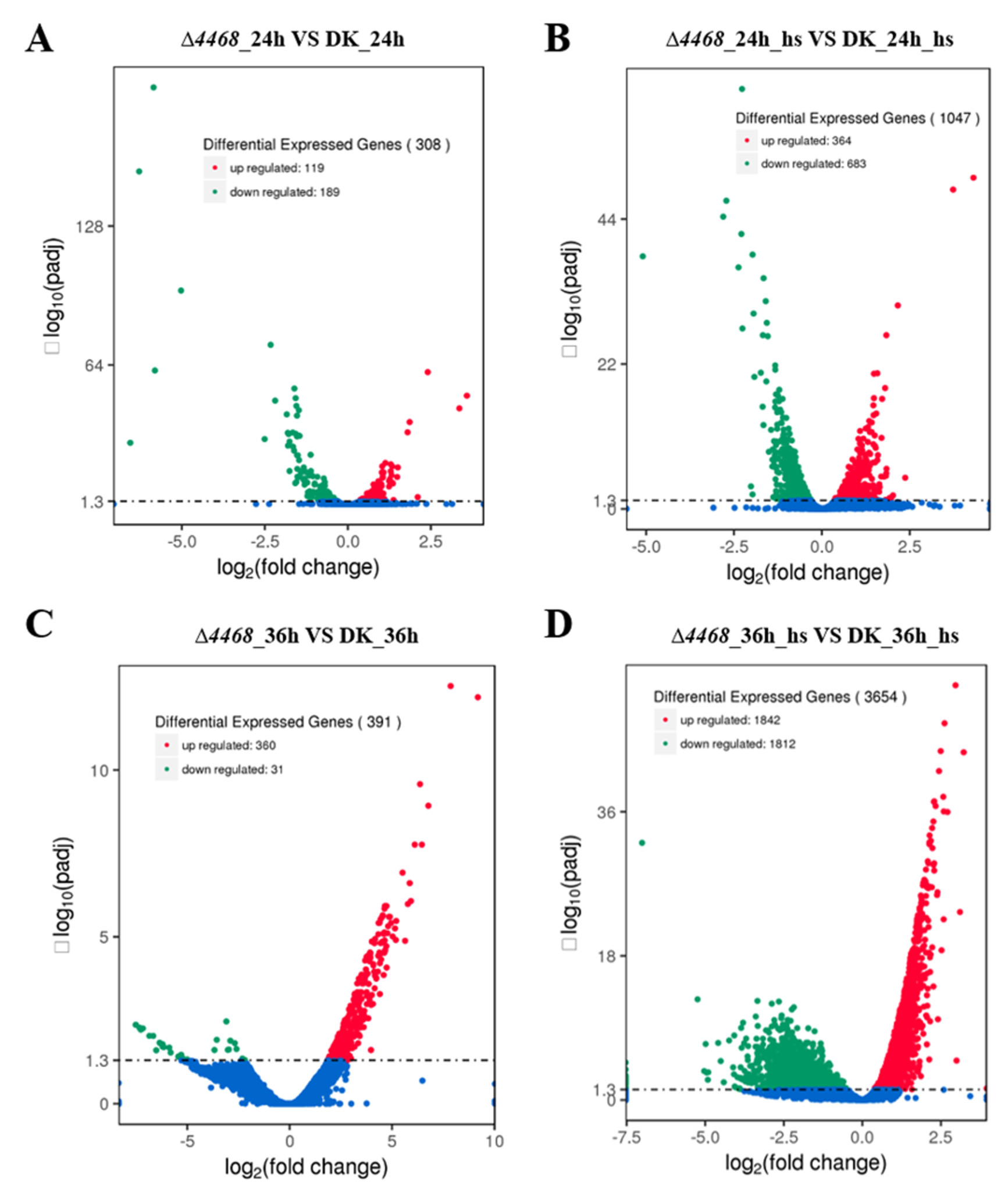

Supplement: FIG S4 [file msystems.01056-21-sf004.tif]

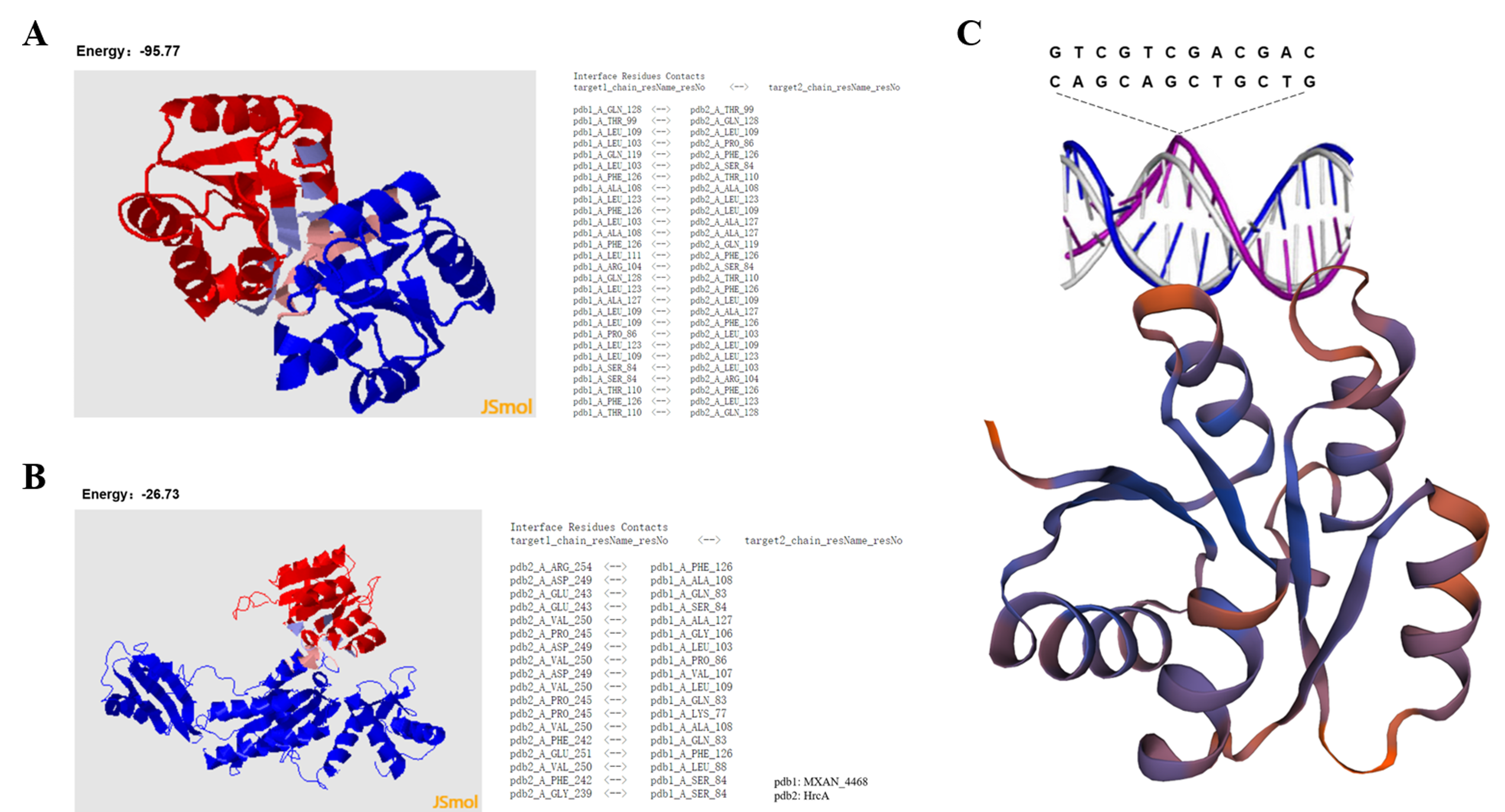

Supplement: FIG S5 [file msystems.01056-21-sf005.tif]
