# Supplementary material for: A Dual-Functional Orphan Response Regulator Negatively Controls the Differential Transcription of Duplicate groELs and Plays a Global Regulatory Role in Myxococcus
Source: mSystems. 2022 Mar 30;7(2):e01056-21. doi: 10.1128/msystems.01056-21 (PMC9040617; doi:10.1128/msystems.01056-21)
Supplement: TABLE S2 [file msystems.01056-21-st002.docx]

**Table S2** Proteins potentially interacting with MXAN_4468, MXAN_4468-D61V and MXAN_4468-D61F in *M. xanthus* DK1622. The proteins interacting with MXAN_4468 that were absent in the results of D61V or D61F are in the colored background.

| **Genes** | **Protein ID** | **Protein Name** | **GO-Molecular Function** |
| --- | --- | --- | --- |
| **Proteins potentially interacting with MXAN_4468** | | | |
| MXAN_0391 | Q1DFB1 | Oxidoreductase | oxidoreductase activity [GO:0016491] |
| MXAN_7494 | Q1CVH6 | tRNA uridine enzyme MnmG | flavin adenine dinucleotide binding [GO:0050660] |
| MXAN_3541 | Q1D6I9 | Succinate--CoA ligase subunit beta | ATP binding [GO:0005524] |
| MXAN_3101 | Q1D7R9 | Glycine--tRNA ligase beta subunit | arginine-tRNA ligase activity [GO:0004814] |
| MXAN_7454 | Q1CVL4 | RNA polymerase sigma-70 factor | DNA binding [GO:0003677] |
| MXAN_4758 | Q1D353 | Chemotaxis protein CheA | phosphorelay sensor kinase activity [GO:0000155] |
| MXAN_7441 | Q1CVM7 | Glycosyl transferase, group 1 | transferase activity [GO:0016757] |
| MXAN_7198 | Q1CWB2 | Uncharacterized protein | metallopeptidase activity [GO:0008237] |
| MXAN_7187 | Q1CWC3 | Glycosyl transferase | transferase activity [GO:0016740] |
| MXAN_7156 | Q1CWF4 | Ribose-phosphate pyrophosphokinase | kinase activity [GO:0016301] |
| MXAN_7059 | Q1CWQ1 | Sensory box histidine kinase | phosphorelay sensor kinase activity [GO:0000155] |
| MXAN_6898 | Q1CX61 | OmpA domain protein | calcium ion binding [GO:0005509] |
| MXAN_6141 | Q1CZA1 | Uncharacterized protein | - |
| MXAN_6964 | Q1CWZ4 | Putative chemotaxis protein CheA | phosphorelay sensor kinase activity [GO:0000155] |
| MXAN_5426 | Q1D1A0 | RNA-splicing ligase RtcB | metal ion binding [GO:0046872] |
| MXAN_4992 | Q1D2H5 | Putative transcriptional regulator | DNA binding [GO:0003677] |
| MXAN_4867 | Q1D2U8 | FHA domain/TonB domain protein | - |
| MXAN_4468 | Q1D3Y4 | Response regulator | phosphorelay signal system [GO:0000160] |
| MXAN_4249 | Q1D4J9 | Uncharacterized protein | - |
| MXAN_3780 | Q1D5W1 | Patatin-like phospholipase protein | hydrolase activity [GO:0016787] |
| MXAN_3571 | Q1D6G2 | Uncharacterized protein | - |
| MXAN_3449 | Q1D6S7 | Heavy metal efflux pump | cation transporter activity [GO:0008324] |
| MXAN_3092 | Q1D7S8 | Serine/threonine kinase family protein | ATP binding [GO:0005524] |
| MXAN_2947 | Q1D866 | Isochorismatase family protein | catalytic activity [GO:0003824] |
| MXAN_2485 | Q1D9G9 | DNA topoisomerase | ATP binding [GO:0005524] |
| MXAN_2258 | Q1DA44 | SNF2/helicase domain protein | ATP binding [GO:0005524] |
| MXAN_1963 | Q1DAX5 | Uncharacterized protein | - |
| MXAN_1816 | Q1DBB1 | Uncharacterized protein | - |
| MXAN_1757 | Q1DBG6 | Transcriptional regulator | DNA binding [GO:0003677] |
| MXAN_1668 | Q1DBQ3 | Serine/threonine kinase protein | kinase activity [GO:0016301] |
| MXAN_1421 | Q1DCE5 | Pseudouridine synthase | pseudouridine synthase activity [GO:0009982] |
| MXAN_1027 | Q1DDI5 | Glycosyl transferase | transferase activity [GO:0016740] |
| MXAN_0907 | Q1DDV5 | Sigma-54 transcriptional regulator | ATP binding [GO:0005524] |
| MXAN_0494 | Q1DF09 | Cation-binding protein | - |
| MXAN_0265 | Q1DFM7 | Serine/threonine protein kinase | ATP binding [GO:0005524] |
| MXAN_6726 | Q1CXN0 | Heat-inducible transcription repressor | DNA binding [GO:0003677] |
| MXAN_0122 | Q1DG18 | Putative GTP cyclohydrolase II | GTP binding [GO:0005525] |
| **Proteins potentially interacting with MXAN_4468 D61V** | | | |
| MXAN_0391 | Q1DFB1 | Oxidoreductase | oxidoreductase activity [GO:0016491] |
| MXAN_7494 | Q1CVH6 | tRNA uridine enzyme MnmG | flavin adenine dinucleotide binding [GO:0050660] |
| MXAN_3541 | Q1D6I9 | Succinate--CoA ligase subunit beta | ATP binding [GO:0005524] |
| MXAN_3101 | Q1D7R9 | Glycine--tRNA ligase beta subunit | arginine-tRNA ligase activity [GO:0004814] |
| MXAN_7198 | Q1CWB2 | Uncharacterized protein | metallopeptidase activity [GO:0008237] |
| MXAN_6898 | Q1CX61 | OmpA domain protein | calcium ion binding [GO:0005509] |
| MXAN_6141 | Q1CZA1 | Uncharacterized protein | - |
| MXAN_4867 | Q1D2U8 | FHA domain/TonB domain protein | - |
| MXAN_4468 | Q1D3Y4 | Response regulator | phosphorelay signal system [GO:0000160] |
| MXAN_4249 | Q1D4J9 | Uncharacterized protein | - |
| MXAN_3780 | Q1D5W1 | Patatin-like phospholipase protein | hydrolase activity [GO:0016787] |
| MXAN_3571 | Q1D6G2 | Uncharacterized protein | - |
| MXAN_3449 | Q1D6S7 | Heavy metal efflux pump | cation transporter activity [GO:0008324] |
| MXAN_2947 | Q1D866 | Isochorismatase family protein | catalytic activity [GO:0003824] |
| MXAN_2485 | Q1D9G9 | DNA topoisomerase | ATP binding [GO:0005524] |
| MXAN_2258 | Q1DA44 | SNF2/helicase domain protein | ATP binding [GO:0005524] |
| MXAN_1963 | Q1DAX5 | Uncharacterized protein | - |
| MXAN_1816 | Q1DBB1 | Uncharacterized protein | - |
| MXAN_1421 | Q1DCE5 | Pseudouridine synthase | pseudouridine synthase activity [GO:0009982] |
| MXAN_1027 | Q1DDI5 | Glycosyl transferase | transferase activity [GO:0016740] |
| MXAN_0494 | Q1DF09 | Cation-binding protein | - |
| MXAN_0265 | Q1DFM7 | Serine/threonine protein kinase | ATP binding [GO:0005524] |
| MXAN_0122 | Q1DG18 | Putative GTP cyclohydrolase II | GTP binding [GO:0005525] |
| **Proteins potentially interacting with MXAN_4468 D61F** | | | |
| MXAN_0391 | Q1DFB1 | Oxidoreductase | oxidoreductase activity [GO:0016491] |
| MXAN_7494 | Q1CVH6 | tRNA uridine enzyme MnmG | flavin adenine dinucleotide binding [GO:0050660] |
| MXAN_3541 | Q1D6I9 | Succinate--CoA ligase subunit beta | ATP binding [GO:0005524] |
| MXAN_3101 | Q1D7R9 | Glycine--tRNA ligase beta subunit | arginine-tRNA ligase activity [GO:0004814] |
| MXAN_7198 | Q1CWB2 | Uncharacterized protein | metallopeptidase activity [GO:0008237] |
| MXAN_6898 | Q1CX61 | OmpA domain protein | calcium ion binding [GO:0005509] |
| MXAN_6141 | Q1CZA1 | Uncharacterized protein | - |
| MXAN_4867 | Q1D2U8 | FHA domain/TonB domain protein | - |
| MXAN_4468 | Q1D3Y4 | Response regulator | phosphorelay signal system [GO:0000160] |
| MXAN_4249 | Q1D4J9 | Uncharacterized protein | - |
| MXAN_3571 | Q1D6G2 | Uncharacterized protein | - |
| MXAN_3449 | Q1D6S7 | Heavy metal efflux pump | cation transporter activity [GO:0008324] |
| MXAN_2947 | Q1D866 | Isochorismatase family protein | catalytic activity [GO:0003824] |
| MXAN_2485 | Q1D9G9 | DNA topoisomerase | ATP binding [GO:0005524] |
| MXAN_2258 | Q1DA44 | SNF2/helicase domain protein | ATP binding [GO:0005524] |
| MXAN_1963 | Q1DAX5 | Uncharacterized protein | - |
| MXAN_1816 | Q1DBB1 | Uncharacterized protein | - |
| MXAN_1421 | Q1DCE5 | Pseudouridine synthase | pseudouridine synthase activity [GO:0009982] |
| MXAN_1027 | Q1DDI5 | Glycosyl transferase | transferase activity [GO:0016740] |
| MXAN_0494 | Q1DF09 | Cation-binding protein | - |
| MXAN_0122 | Q1DG18 | Putative GTP cyclohydrolase II | GTP binding [GO:0005525] |
