# Supplementary material for: A Dual-Functional Orphan Response Regulator Negatively Controls the Differential Transcription of Duplicate groELs and Plays a Global Regulatory Role in Myxococcus
Source: mSystems. 2022 Mar 30;7(2):e01056-21. doi: 10.1128/msystems.01056-21 (PMC9040617; doi:10.1128/msystems.01056-21)
Supplement: TABLE S3 [file msystems.01056-21-st003.docx]

**Table S****3** Comparison of the reads of transcriptomic data and reference genomes

| **Sample name** | **DK_24h_1** | **DK_24h_2** | **DK_24h_3** | Δ***4468*_24h_1** | Δ***4468*_24h_2** | Δ***4468*_24h_3** | **DK_24h_hs_1** | **DK_24h_hs_2** | **DK_24h_hs_3** | **_24h_hs_1** | **_24h_hs_2** | **_24h_hs_3** |
| --- | --- | --- | --- | --- | --- | --- | --- | --- | --- | --- | --- | --- |
| **Total reads^1^** | 19562870 | 22021536 | 19026938 | 15962678 | 15705198 | 17877506 | 18481954 | 17958994 | 21407108 | 12326120 | 17102540 | 16349810 |
| **Total mapped^2^** | 19450357 (99.42%) | 21877930 (99.35%) | 18905541 (99.36%) | 15876283 (99.46%) | 15593381 (99.29%) | 17737660 (99.22%) | 18353632 (99.31%) | 17807984 (99.16%) | 21239991 (99.22%) | 12232780 (99.24%) | 16974419 (99.25%) | 16223253 (99.23%) |
| **Multiple mapped^3^** | 496317 (2.54%) | 625934 (2.84%) | 542145 (2.85%) | 375131 (2.35%) | 456164 (2.9%) | 506059 (2.83%) | 537953 (2.91%) | 505819 (2.82%) | 666055 (3.11%) | 346557 (2.81%) | 474485 (2.77%) | 496859 (3.04%) |
| **Uniquely mapped^4^** | 18954040 (96.89%) | 21251996 (96.51%) | 18363396 (96.51%) | 15501152 (97.11%) | 15137217 (96.38%) | 17231601 (96.39%) | 17815679 (96.39%) | 17302165 (96.34%) | 20573936 (96.11%) | 11886223 (96.43%) | 16499934 (96.48%) | 15726394 (96.19%) |

| **Sample**  **name** | **DK_36h_1** | **DK_36h_2** | **DK_36h_3** | Δ***4468*_36h_1** | Δ***4468*_36h_2** | Δ***4468*_36h_3** | **DK_36h_hs_1** | **DK_36h_hs_2** | **DK_36h_hs_3** | Δ***4468*_36h_hs_1** | Δ***4468*_36h_hs_2** | Δ***4468*_36h_hs_3** |
| --- | --- | --- | --- | --- | --- | --- | --- | --- | --- | --- | --- | --- |
| **Total reads^1^** | 20469482 | 18226142 | 18308128 | 15420978 | 15367126 | 16155138 | 13823416 | 16669824 | 18624486 | 16019988 | 18916810 | 18919588 |
| **Total mapped^2^** | 20344338 (99.39%) | 18083463 (99.22%) | 18170012 (99.25%) | 15308106 (99.27%) | 15238454 (99.16%) | 16019422 (99.16%) | 13699772 (99.11%) | 16534326 (99.19%) | 18498932 (99.33%) | 15874975 (99.09%) | 18778027 (99.27%) | 18800528 (99.37%) |
| **Multiple mapped^3^** | 420894  (2.06%) | 507720 (2.79%) | 491211 (2.68%) | 429496 (2.79%) | 441386 (2.87%) | 445698 (2.76%) | 457414 (3.31%) | 476187 (2.86%) | 515353 (2.77%) | 429727 (2.68%) | 553428 (2.93%) | 476000 (2.52%) |
| **Uniquely mapped^4^** | 19923444 (97.33%) | 17575743 (96.43%) | 17678801 (96.56%) | 14878610 (96.48%) | 14797068 (96.29%) | 15573724 (96.4%) | 13242358 (95.8%) | 16058139 (96.33%) | 17983579 (96.56%) | 15445248 (96.41%) | 18224599 (96.34%) | 18324528 (96.85%) |

1. Total reads: the number of sequencing fragments filtered by sequencing data (Clean data). 2. Total mapped: the number of sequencing fragments that can be located on the reference genome. 3. Multiple mapped: the number of sequencing fragments with multiple alignment positions on reference sequences. 4. Uniquely mapped: the number of sequencing fragments with unique alignment positions on reference sequences.
