# Supplementary material for: A Dual-Functional Orphan Response Regulator Negatively Controls the Differential Transcription of Duplicate groELs and Plays a Global Regulatory Role in Myxococcus
Source: mSystems. 2022 Mar 30;7(2):e01056-21. doi: 10.1128/msystems.01056-21 (PMC9040617; doi:10.1128/msystems.01056-21)
Supplement: TABLE S4 [file msystems.01056-21-st004.docx]

**Table S4** Bacterial strains, plasmids and primers used in this study

Strains and plasmids:

| **Strain and plasmid** | **Genotype or description** | **Source or reference** |
| --- | --- | --- |
| **Strains** | | |
| *M. xanthus* | | |
| DK1622 | Wild-type strain | D.Kaiser University of Standford |
| Δ*4468* | DK1622 Δ*MXAN_4468* | This study |
| Δ*4468*Δ*hrcA* | DK1622 Δ*MXAN_4468* Δ*MXAN_6726* | This study |
| KI68-1 | DK1622 knockout 185 bp fragment of *MXAN_4468* | This study |
| KI68-2 | DK1622 knockout 199 bp fragment near *MXAN_4468* | This study |
| KI68-3 | DK1622 knockout 193 bp fragment near *MXAN_4468* | This study |
| KI68-4 | DK1622 knockout 685 bp fragment near *MXAN_4468* | This study |
| Δ*4468*::*4468* | DK1622 Δ*MXAN_4468* :: *MXAN_4468* | This study |
| DK::P*_pilA_*+*4468* | DK1622 Δ*MXAN_4468* :: P*_pilA_*+*MXAN_4468* | This study |
| Δ*4468*::*4468-*D61V | DK1622 Δ*MXAN_4468* :: *MXAN_4468*（61^st^，GTC） | This study |
| Δ*4468*::*4468-*D61F | DK1622 Δ*MXAN_4468* :: *MXAN_4468*（61^st^，TTC） | This study |
| Δ*6964* | DK1622 Δ*MXAN_6964* | This study |
| Δ*4758* | DK1622 Δ*MXAN_4758* | This study |
| Δ*6692* | DK1622 Δ*MXAN_6692* | This study |
| DK1622-km | DK1622 strain site-specific integrated by pSWU19; Km^r^ | This study |
| Δ*4468*-km | Δ*4468* mutant strain site-specific integrated by pSWU19; Km^r^ | This study |
| *E. coli* | | |
| BL21(DE3) | F^-^ompT hsdS_B_(r_B_^-^m_B_^-^)gal dcm (DE3) | Stratagene |
| XL1-Blue MR | Δ(mcrA)183Δ(mcrCB-hsdSMR-mrr)173 endA1 supE44 thi-1 recA1 gyrA96 relA1 lac | Stratagene |
| **Plasmids** | | |
| pBJ113 | Gene replacement vector with KG cassette; Km^r^ | Laboratory collection |
| pSWU19 | Site-specific integration vector with Mx8 attB integration site; Km^r^ | Laboratory collection |
| pET32a | Expression vector, Amp^r^, N-His·tag, C-His·tag, T7 promotor | Laboratory collection |
| *4468*-pET32a | *MXAN_4468* insertion in pET32a by EcoRⅠ/ XhoⅠ | This study |
| pMAL-c5X | Amp^r^，Tac promoter，MBP Tag | Laboratory collection |
| *4468*-pMAL-c5X | *MXAN_4468* insertion in pMAL-c5X by NdeⅠ/ BamHⅠ | This study |
| *hrcA*-pMAL-c5X | *hrcA* insertion in pMAL-c5X by NdeⅠ/ BamHⅠ | This study |

Primers:

| **Primer** | **Sequence (5-3)*** | **Use** |
| --- | --- | --- |
| 4468-KO-UF | TGGTGACGTTGTCCAGGTACTGC | Amplification of upstream homologous arm for deletion of *MXAN_4468* |
| 4468-KO-UR | GAGGTGCGCCGTGGGGGAACGTAGCAGCCGTGCG |  |
| 4468-KO-DF | CGCACGGCTGCTACGTTCCCCCACGGCGCACCTC | Amplification of downstream homologous arm for deletion of *MXAN_4468* |
| 4468-KO-DR | GGAGCGGGCCACCTGCT |  |
| 4468-OE-UF | GCTCTAGACCGCGACCCTGTCGGATT | PCR amplification of *pilA* promotor for fusion with *MXAN_4468* |
| 4468-OE-UR | GCACCAGGACGGGCCAAGGGGGTCCTCAGAGAAGGTT |  |
| 4468-OE-DF | AACCTTCTCTGAGGACCCCCTTGGCCCGTCCTGGTGC | PCR amplification of *MXAN_4468* |
| 4468-OE-DR | GGAATTCCTATTGCGCGAAGCGGACC |  |
| 4468-67-P1-UP | GGTCCGCTTCGCGCAATAG | PCR amplification of the *MXAN_4468-groEL2* locus（P1） |
| 4468-67-P1-DOWN | CTGGGGGATTTTACTAGTACGACAC |  |
| 4468-67-P2-UP | GGTCCGCTTCGCGCAATAG | PCR amplification of the *MXAN_4468-groEL2* locus（P2） |
| 4468-67-P2-DOWN | GCGCGGACTGATGGAAGAAAA |  |
| 4468-GTC-UP | CATCACCGTCTACGTGATGCCCCATATGGACG | PCR amplification of *MXAN_4468*（61^st^，GTC） |
| 4468-GTC-DOWN | TCACGTAGACGGTGATGACCATGCTGACCTGG |  |
| 4468-TTC-UP | CATCACCTTCTACGTGATGCCCCATATGGACG | PCR amplification of *MXAN_4468*（61^st^，TTC） |
| 4468-TTC-DOWN | TCACGTAGAAGGTGATGACCATGCTGACCTGG |  |
| EcoRⅠ-4468-up | GGAATTCATGGCCCGTCCTGGTGC | PCR amplification of *MXAN_4468* for construction of 4468-pET32a |
| XhoⅠ-4468-down | CCGCTCGAGCTATTGCGCGAAGCGGACC |  |
| NdeⅠ-4468-up | GGAATTCCATATGATGGCCCGTCCTGGTGC | PCR amplification of *MXAN_4468* for construction of 4468-pMAL-c5X |
| BamHⅠ-4468-down | CGGGATCCCTATTGCGCGAAGCGGACC |  |
| groEL1-Q-up | CAAGGACGGTGTGACGGTCG | Transcriptional level detection of *groEL1* |
| groEL1-Q-down | CCTTGTCGATGCCGCGCTTG |  |
| groEL2-Q-up | GAAGAGCTTCGGCTCCCCCA | Transcriptional level detection of *groEL2* |
| groEL2-Q-down | TTGAGGTCCATCGGGCTGTG |  |
| 4468-Q-up | AGTCCCGTGAACATCCTTGTCG | Transcriptional level detection of *MXAN_4468* |
| 4468-Q-down | CGCCTTGAGCATCTCCGTG |  |
| groES-Q-up | TCGCCGAGGAGAACAAGACC | Transcriptional level detection of *groES* |
| groES-Q-down | CACGGAGGATGAGGTGCTCCT |  |
| gapA-Q-up | GCCCTGGAAGAGCCTGAACG | Reference gene for transcriptional level detection |
| gapA-Q-down | TCGAGATGACGTGGTGCTTGG |  |
